# Energy dissipation in functionally two-dimensional phase transforming cellular materials

**DOI:** 10.1038/s41598-019-48581-8

**Published:** 2019-08-29

**Authors:** Yunlan Zhang, David Restrepo, Mirian Velay-Lizancos, Nilesh D. Mankame, Pablo D. Zavattieri

**Affiliations:** 1 Lyles School of Civil Engineering, Purdue University, West Lafayette, IN 47907 USA; 2 Vehicle Systems Research, General Motors Global Research & Development, Warren, MI 48090 USA; 3 Present Address: Department of Mechanical Engineering, The University of Texas at San Antonio, San Antonio, TX 78249 USA

**Keywords:** Mechanical properties, Mechanical engineering

## Abstract

Phase Transforming Cellular Materials (PXCMs) are periodic cellular materials whose unit cells exhibit multiple stable or meta-stable configurations. Transitions between the various (meta-) stable configurations at the unit cell level enable these materials to exhibit reusable solid state energy dissipation. This energy dissipation arises from the storage and non-equilibrium release of strain energy accompanying the limit point traversals underlying these transitions. The material deformation is fully recoverable, and thus the material can be reused to absorb and dissipate energy multiple times. In this work, we present two designs for functionally two-dimensional PXCMs: the *S-type* with four axes of reflectional symmetry based on a square motif and, the *T-type* with six axes of symmetry based on a triangular motif. We employ experiments and simulations to understand the various mechanisms that are triggered under multiaxial loading conditions. Our numerical and experimental results indicate that these materials exhibit similar solid state energy dissipation for loads applied along the various axes of reflectional symmetry of the material. The specific energy dissipation capacity of the *T-type* is slightly greater and less sensitive to the loading direction than the *S-type* under the most of loading directions. However, both types of material are shown to be very effective in dissipating energy.

## Introduction

Plastic deformation of cellular materials such as metal foams and honeycombs is commonly used for absorbing and dissipating energy because these materials can absorb large amounts of energy per unit mass^1^. However, the deformation in these cases is irreversible and hence, the material can only be used for a single energy absorption event. Phase Transforming Cellular Materials (PXCMs) are a class of periodic cellular materials that exhibit solid state energy absorption and dissipation^2^ and are comparable to honeycombs, especially at low plateau stresses. Moreover, PXCMs can be used multiple times as they do not rely on irreversible deformation of their base material for energy dissipation. The elementary building blocks of PXCMs exhibit multiple stable or meta-stable configurations^2–4^. Each stable or metastable configuration defines a phase at the building blocks level, and the transitions between these building block configurations can be interpreted as phase transformations. The ability of these materials to exhibit reversible solid state energy dissipation arises from the storage and subsequent non-equilibrium release of strain energy accompanying the limit point traversals underlying these transitions. The mechanical response of an ensemble of building blocks shows separate loading and unloading plateaus that are characteristic of solid state phase transformations in NiTi alloys^5^ and configurational changes in biological shock absorbers like the protein titin in sarcomeres^6,7^.

PXCMs have been proposed for applications such reusable, solid state energy absorption^2,8,9^, shock or impact isolation^10,11^, and reconfigurable structures^12,13^. PXCMs have also been used to create metamaterials whose mechanical properties (e.g. compressive modulus, wave propagation behavior) can be altered after the material has been fabricated^8,10,14^. Many of the PXCMs cited above are structurally two or three-dimensional materials because they can resist loads applied along arbitrary directions in the plane and space respectively. However, they are functionally one-dimensional materials because they exhibit significant solid state energy dissipation only for loads applied along a preferred loading direction. Some recent work addresses materials that exhibit solid state energy dissipation for loads applied along multiple directions. Straightforward extensions of the functionally one dimensional PXCM design to two and three dimensions have been proposed by Shan *et al*. and Ren *et al*.^15,16^. Topology optimization based automatic synthesis techniques have been used to generate functionally two dimensional PXCM unit cells^17^. A two dimensional PXCM unit cell featuring a 5-bar planar truss and a three dimensional tetrahedral PXCM unit cell have been proposed recently^18^. Also, some of the material designs proposed for auxetic materials^19^ and shape reconfigurable materials^13^ have the potential to be functionally two and three dimensional PXCMs. The Miura-ori pattern based metamaterials^20^, three dimensional arrays of spherical shells with patterned holes or Bucklicrystals^21^ and the tape based three dimensional multistable structures^22^ are other examples of materials that might function as two or three dimensional PXCMs. However, in most of the above works the emphasis has either been on the one-dimensional variant of the design or on the response of the higher dimensional material designs to uniaxial loads applied along one direction. In this paper, we study two designs for functionally two-dimensional PXCMs. The first design is based on a square motif and exhibits four axes of reflectional symmetry (similar to^15–17^). The second design is based on a triangular motif and has six axes of symmetry

## Design Considerations

**Figure 1:**
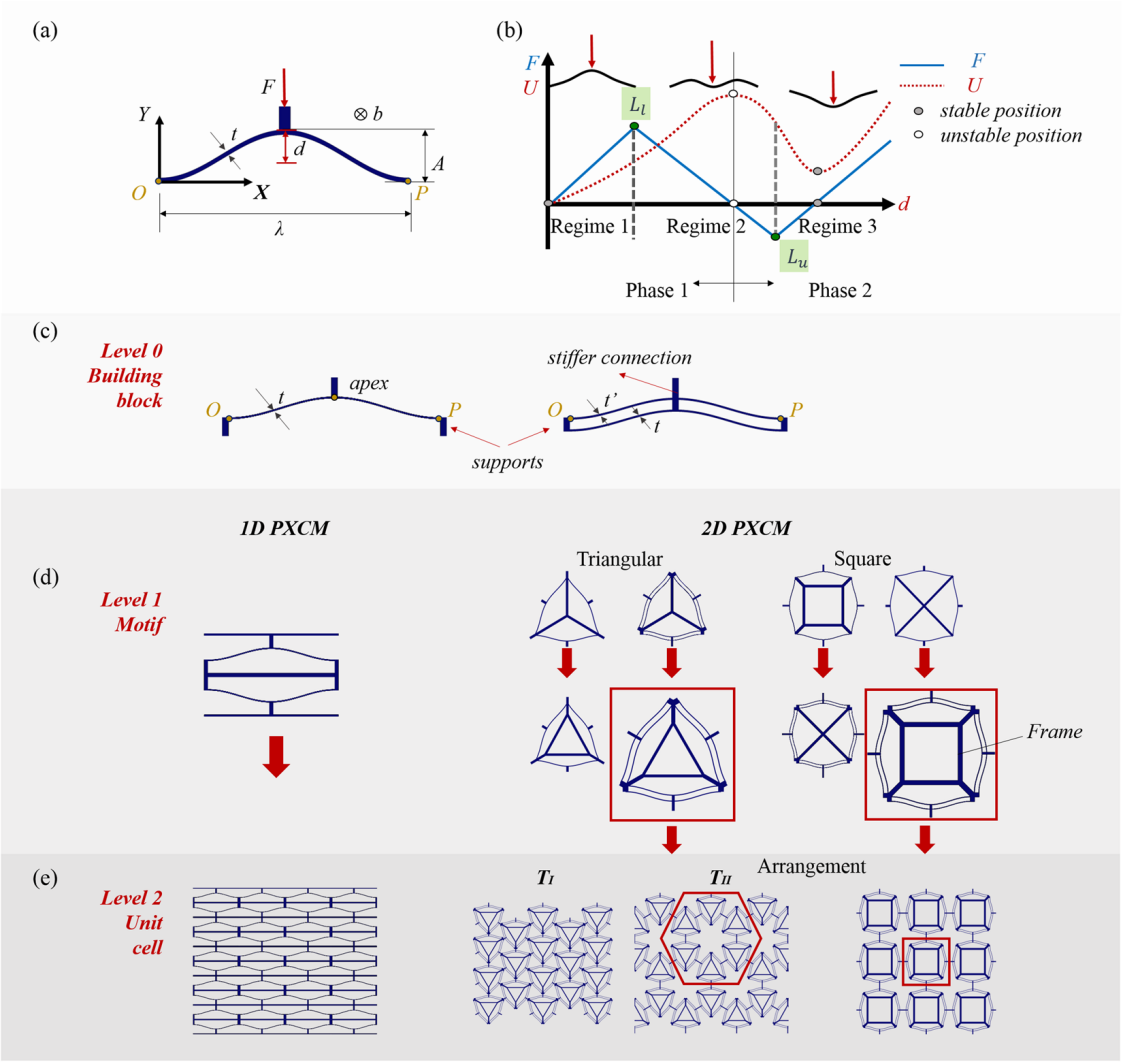
Hierarchical construction of functionally two-dimensional PXCMs. (**a**) Geometry of the elementary bent beam. (**b**) Schematic representation of the force-displacement (*F-d*) and energy-displacement (*U-d*) response of a bistable bent beam. (**c**–**e**) Levels 0–2 of the hierarchical structure of the 2D PXCMs studied in this work.

We first describe how multiple instances of the elementary bistable beam mechanism, considered as the basic building block, can be arranged to create several functionally two-dimensional PXCMs. The shape of the elementary beam is that of the first buckling mode of a straight prismatic beam under axial loading (Fig. 1(a)), represented by $Y=\,(A/2)[1\,-\,cos(2\pi X/\lambda )$], where *A* is the peak to valley amplitude and *λ* is the wavelength. A concentrated load *F* is applied to the beam at its apex, orthogonal to the line *OP* that joins the two ends of the beam, and the resulting displacement *d* at the apex of the beam is noted.

The mechanical response for this beam is shown schematically in Fig. 1(b). The total internal energy *U* (red dashed line) has three extremal points: (1) a stable global minimum in the undeformed configuration (*d* = 0, *F* = 0), (2) an unstable maximum at (*d* = *A*, *F* = 0), and 3) a stable local minimum at (*d* > A, *F* = 0). The force-displacement response (solid blue line) has two positive stiffness branches that are separated by a negative stiffness branch. Two elastic limit points (*L*_*l*_ for increasing loads and *L*_*u*_ for decreasing loads) mark the ends of the negative stiffness branch. They also divide the *F-d* response into three regions as shown in Fig. 1(b). Strain energy is stored in the structure during the deformation from the undeformed configuration to *L*_*l*_. If the structure is loaded beyond this point, it ‘snaps-through’ under force-control to the point on the second positive stiffness branch that can support the same load. This snap-through is associated with non-equilibrium release of some of the stored strain energy which gives PXCMs their ability to dissipate energy without undergoing irreversible deformation. A similar behavior is observed during unloading when the structure snaps-through under force control from *L*_*u*_ to the point on the first positive stiffness branch that can support the same load.

The dimensionless parameter *Q* = *A*/*t* governs the number of stable configurations for this structure as follows^23,24^:

1. Metastable: If *Q* < 2.31, the mechanism has only one stable configuration at (*d* = 0, *F* = 0) as the configuration corresponding to the third extremal point (*d* > *A*) does not persist when the applied load is removed.
2. Bistable: If *Q* ≥ 2.31, the mechanism has two stable configurations as discussed earlier. The mechanism can persist indefinitely in either of the stable configurations when the external load is removed.

In the interval *Q* ϵ [2.3, 2.41], the behavior of the mechanism is sensitive to small variations in the geometry parameters, and hence cannot be characterized in a robust manner^2^.

Qiu *et al*. proposed an alternative design that uses two parallel bent beams (see Fig. 1(c)) that can improve the range over which the bent beam mechanism exhibits bistable behavior^23,24^. The stiffer connection between the two beams at their apexes mitigates the tendency of the single beam to rotate at that location in the absence of an external rotational constraint. This suppresses an asymmetric mode that allows the single beam mechanism to revert back to its undeformed configuration when the external load is removed. This design adds two more geometry parameters: the thickness *t*’ of the top sinusoidal beam and the spacing *s* between the two beams (See Fig. S1; in the supplementary material). The parallel bent beam mechanism can be used instead of, or in conjunction with the single bent beam mechanism discussed earlier.

Figure 1c–e show how 1D and 2D PXCMs can be constructed in a hierarchical manner beginning with the elementary beam structures shown in Fig. 1a. These building blocks comprising one or more elementary bent beam structures constitute the zeroth level of the hierarchy (Fig. 1(c)) . In addition to these bent beam structures, the building blocks also include supports which are much stiffer than the bent beams. These structural supports need to be sufficiently stiff so that the elementary beam structures can exhibit the limit point traversal behavior that is essential for energy dissipation, without adding too much mass into the system. Triangular and square motifs are natural candidates for building the *first* hierarchical level of 2D PXCMs (Fig. 1(d)) because of they can be tessellated to cover a plane^25,26^. However, the choice of the support structures for these building blocks is not straightforward. Two alternative support structure topologies are shown in Fig. 1(d) (Fig. S2). The bulk 2D PXCM material constitutes the *second* level of the hierarchy. The square motif can be tiled only in one way as shown in Fig. 1(e)^17^. However, the triangular motif can be arranged in two different ways as shown in Fig. 1(e). The regular tiling (${T}_{I}$) does not lead to a functionally two-dimensional PXCM (see Fig. S3). The arrangement shown in (${T}_{II}$) with triangular motifs located at the nodes of a regular hexagon is not a tiling as it includes some empty space at the center of the hexagon. However, this arrangement yields a functionally 2D PXCM. The *third* level of this hierarchy would comprise a structural member that is made of the 2D PXCM, but is not shown in Fig. 1.

While several combinations of the above design choices are possible, only some of these lead to robust functionally two-dimensional PXCMs. If the rotation at the apex of the single bent beam mechanisms is not restrained, ensembles of such mechanisms may exhibit local ‘wobble’ modes that give rise to unpredictable and disorderly transformation behavior (see Fig. S1). On the other hand, the 2D PXCMs with the parallel bent beam mechanisms do not suffer from this drawback. They also exhibit higher energy dissipation and a more stable deformation behavior than the corresponding 2D PXCM comprising single bent beam mechanisms. For the same mass of the support structures, the designs with just spokes are much less stiff than the designs with a central frame and spoke construction (see Fig. S2). The latter support topology also gives rise to a higher energy dissipation and repeatable material behavior for the reasons discussed above. Hence, we use the frame and spoke support topology for the designs in this work (See Video 8 in the supplementary material). When every bent beam in an ensemble of bent beam mechanisms transitions from the first stable configuration (regime 1 of the mechanical response as shown in Fig. 1(b)) to the second stable (or metastable) configuration (regime 3), the topology of the building blocks remains unchanged but there is a cooperative rearrangement of the elements of the block. This is reminiscent of the non-diffusive rearrangement of atoms in solid state displacive phase transformations, e.g. in nearly equiatomic NiTi alloys. Many phase changes in physical systems are associated with a discontinuous change in the first derivative of a state variable. In PXCMs, there is a step change in the specific volume of the ensemble because the beams are packed more closely together in the second (meta-)stable configuration than in the first stable configuration. This analogy leads to the following interpretation for PXCMs. When all bent beams in a part of a PXCM sample are in regimes 1 or 3, that part of the PXCM is said to be in phase 1 or 2 respectively (Fig. 1(b)). Any intermediate stage when some beams in a part of a PXCM are in phase 1 and others are in phase 2, the part of the PXCM is deemed to be a mixture of phases. The transformation from phase 1 to phase 2 is referred to as the *forward phase transformation*, while that from phase 2 to phase 1 is called the *reverse phase transformation*.

**Figure 2:**
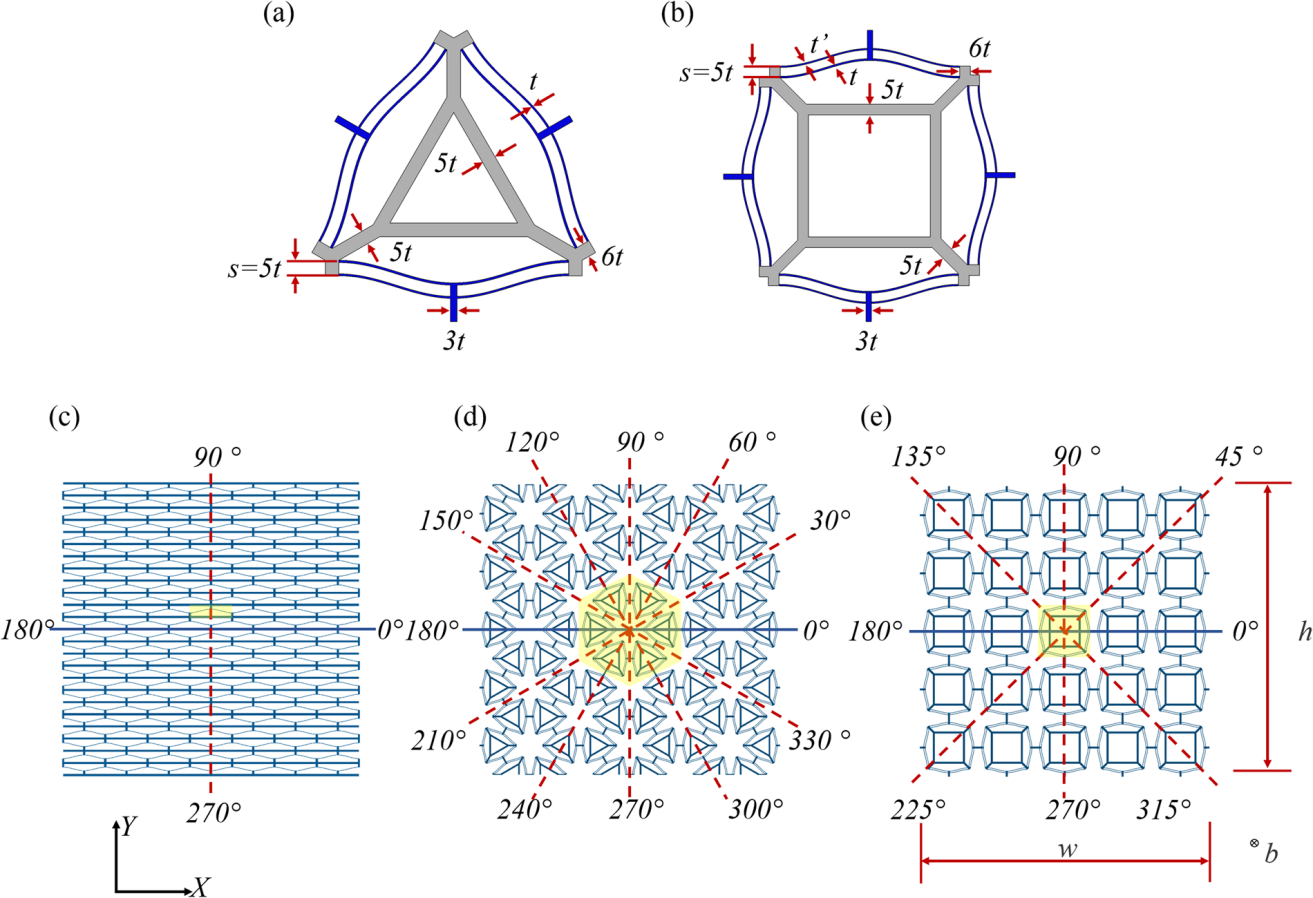
Geometry of the (**a**) *T-type* and (**b**) *S-type* PXCMs motifs. (**c**) 1D PXCM has two axes of symmetry at {0°, 90°}, (**d**) the *T-type* 2D PXCM have six axes of symmetry at {0°, 30°, 60°, 90°, 120°, 150°}, and (**e**) the *S-type* 2D PXCM has four axes of symmetry at {0°, 45°, 90°, 135°}.

The geometries of the two motifs – the square shaped *S-type* and the triangular *T-type* – are shown in Fig. 2. The values assigned to the various design parameters are summarized in Table S1. These parameter values are selected such that the base material remains in the elastic regime while undergoing phase transition over multiple loading cycles^23,24^. The axes of reflectional symmetry for the materials are overlaid in red dashed lines in Fig. 2. In this paper, axes of symmetry refer to planes of symmetry in a three-dimensional setting. The *T-type* motif has three axes of symmetry, the 2D PXCMs unit cell comprising these motifs have six axes of symmetry (see Fig. 2(d)). Three of these are derived from those of the motifs and the other three arise from the hexagonal arrangement of the motifs to form the PXCMs. The *S-type* PXCM and its motifs, both, have four axes of symmetry (see Fig. 2(e)).

## Results

Uniaxial, quasi-static, compressive load-unload tests are used to characterize the response of the *S-* and *T-* type PXCMs along the various axes of symmetry for the materials. These load cases are then recreated using nonlinear finite element analyses. More complex two-dimensional load cases such as bi-axial loading, bending and indentation are outside the scope of this paper and will be addressed in a future publication. In the figures that follow, the coordinate system {*a*_1_*,a*_2_} is embedded in the material sample. This rotates relative to the fixed global coordinate system {*X, Y*} as the samples are loaded along different axes. Material symmetries render loading along some of the axes of symmetry to be equivalent to loading along other axes. This reduces the number of load cases that need to be considered in this study.

**Figure 3:**
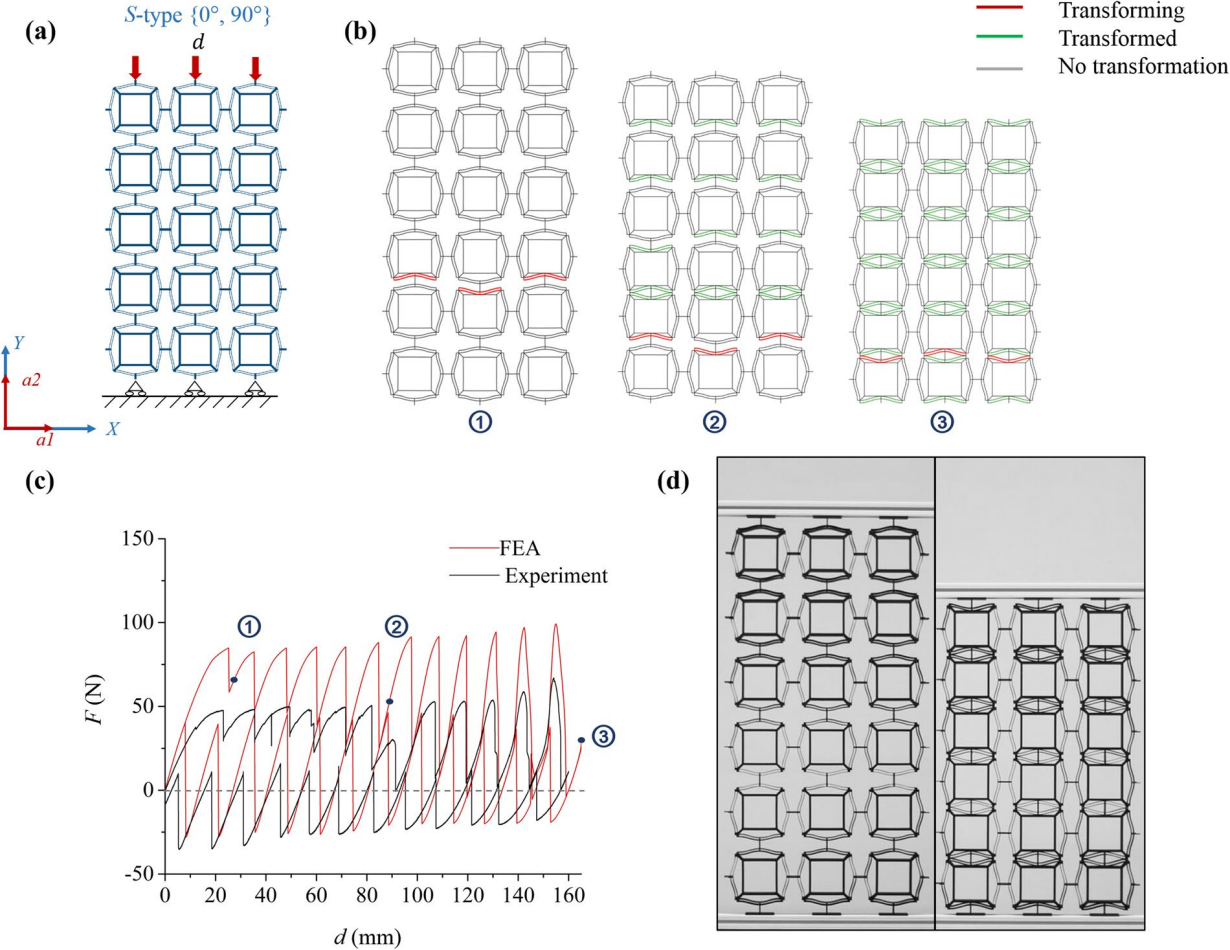
Performance of a *S-type* PXCM sample under one compressive load-unload cycle at {0°, 90°}. (**a**) The sample is under uniaxial loading condition and supported by rollers at bottom. (**b**) Phase transformation sequence of the three characteristic states from FE simulation. (**c**) *F-d* relation of sample from FE simulation and Experiment. (d) The states of the sample at initial and final deformed configurations (See Videos 1 and 7 in the supplementary material).

Figure 3 summarizes the mechanical response of a functionally two-dimensional *S-type* PXCM. The schematic for this load case is shown in Fig. 3(a) where the *a*_1_ axis is aligned with (loading at 0°) or orthogonal to (loading at 90°) the X-axis. Figure 3(b) showed three characteristic states during the phase transformation process corresponding to three labeled points in Fig. 3(c). The *F-d* responses from the finite element simulation (solid red line) and the experiment (solid black line) are overlaid in Fig. 3(c). The experimental *F-d* curves for three cycles are presented in Fig. 5 S(a). The initial (undeformed) and final configurations from the experiment are shown in Fig. 3(d).

The *F-d* response obtained from the finite element simulations agrees qualitatively with the experimentally recorded one. The finite element and experimental responses show serrated loading and unloading plateaus as expected and shown in 1D PXCM^2^. There are 12 peaks in both branches which correspond to the number of bent beam elements in any column of motifs in the sample. Each peak marks the transformation of a bent beam element from phase 1 to phase 2. All cells in this sample exhibit bistable behavior as indicated by the negative compressive (i.e. tensile) force needed to revert the sample back to phase 1. The key quantitative differences between the FE simulations and experimental results are a) the experimental loading and unloading branches lie below the corresponding ones from the finite element simulations and b) one peak in the loading branch of the experimental response is lower than the others. Figure 3(b) shows the deformed configurations of the sample at three salient points during its compression as obtained from the finite element simulations. The corresponding points are labelled on the *F-d* response in Fig. 3(c). The bent beams are color coded according to their status at that point in the deformation. The beams rendered in gray are still in phase 1, those shaded green have already transformed to phase 2, and the red ones are undergoing phase transformation. Identification of the beams that are either in phase 1 or 2 is straightforward. However, determining which beams are in the process of phase transformation is challenging because the limit point traversal (or snap-through) is a non-equilibrium event that occurs quickly and the finite element solution is unable to follow it consistently. We circumvent this difficulty by inspecting the states of the sample at the solution points just before and just after the salient point under consideration. Any beams that have changed phases between these two neighboring points are deemed to be undergoing phase transformation at this salient point, and are shaded red in Fig. 3(b).

The locations and distribution of the red beams in Fig. 3(b) serve as indicators for the nucleation and propagation of the phase transformations. Note that the beams undergoing phase transformation at these salient points lie along a (horizontal) row in the sample. This indicates that even though the different rows may transform at different levels of global compressive strain, an entire row transforms together. This ensures that the loss of stability is restricted to one row of beams and the rest of the sample remains stable. In turn, this leads to an orderly progressive collapse of the different bent beam rows as is evident from the regular shapes of the serrations in the *F-d* response. We observe that only the bent beams that are approximately parallel to the *a*_1_ (*a*_2_ for the 90° loading case) axis of the material have undergone phase transformation at the end of the deformation process. Those that are approximately parallel to *a*_2_ (*a*_1_ for the 90° loading case) axis remain in phase 1. Thus, a fairly significant fraction of the bent beams in the PXCM did not contribute to the energy dissipation in this case.

**Figure 4:**
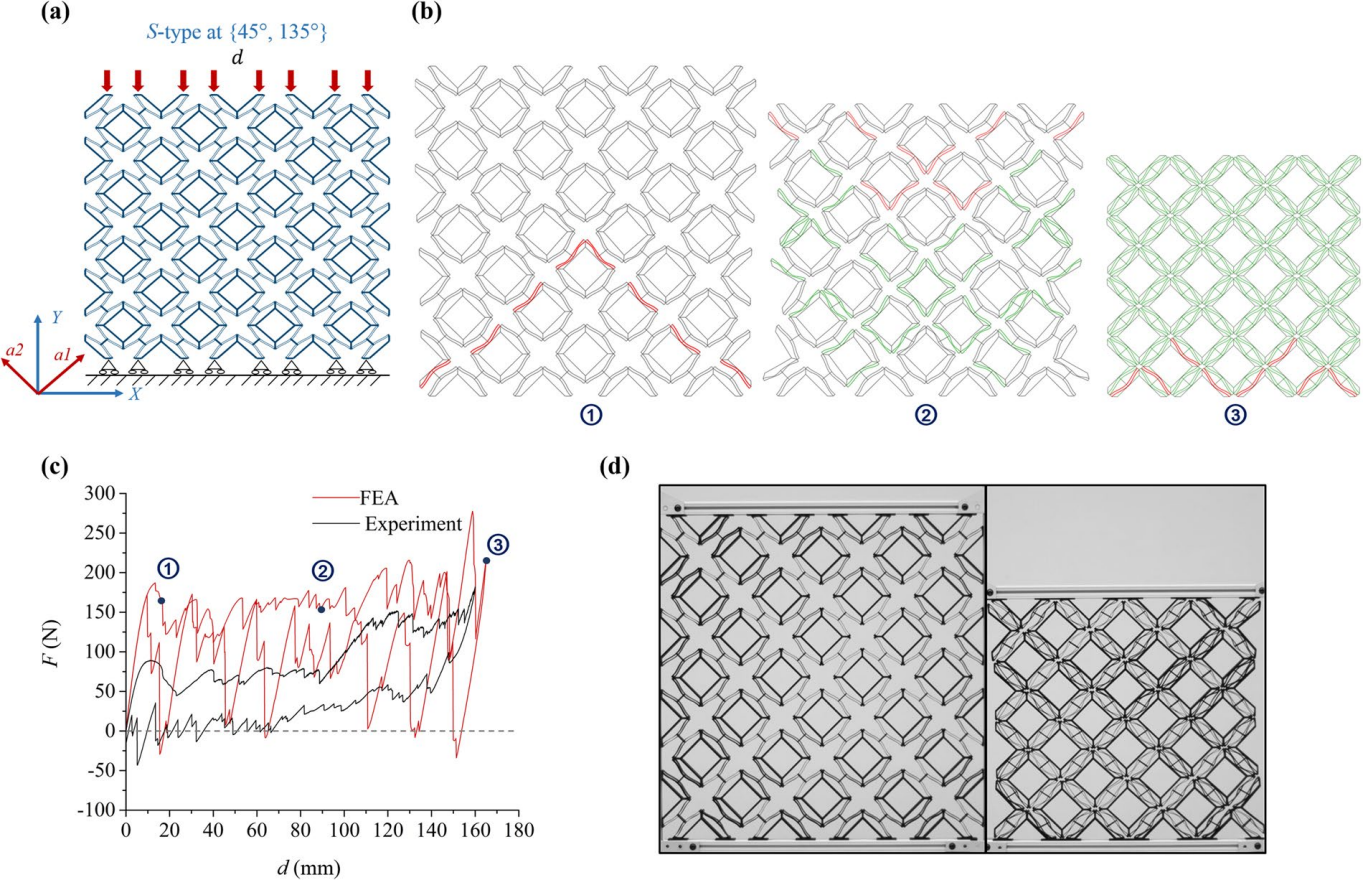
Performance of a *S-type* PXCM sample under one compressive load-unload cycle at {45°, 135°}. (**a**) The sample is under uniaxial loading condition and supported by rollers at bottom. (**b**) Phase transformation sequence of the three characteristic states from FE simulation. (**c**) *F-d* relation of sample from FE simulation and Experiment. (**d**) The states of the sample at initial and final deformed configurations (See Videos 2 and 7 in the supplementary material).

Figure 4 summarizes the response of a *S-type* PXCM with the square shaped motifs when the ${a}_{1}$ axis of the material is inclined at 45° or 135° to the global X-axis. The various sub-figures for Fig. 4 are similar to those for Fig. 3. Figure 4(c) shows qualitative agreement between the experimental and simulated responses. Both responses show loading and unloading plateaus that are separated from each other. However, neither of these responses show a serrated pattern that as distinct as in the previous ({0°, 90°}) case. Also, we note that the simulated response in Fig. 4(c) shows a PXCM with some mechanisms exhibiting bistable behavior especially near the beginning of the load and unload branches. However, the experimental response is that of a metastable PXCM except at the end of the unloading branch. Recall that the same material behaved like a bistable PXCM for the {0°, 90°} load case. Both of these observations relate to the fact that the ${a}_{1}$ axis of the material is now inclined to the applied displacement. As it will be discussed later, the resulting asymmetry reduces the snapping action of the mechanisms changing the nature of the individual transitions, leading to smoother serration in the *F-d* response. Unlike the previous case, all of the bent beams have transformed to phase 2 by the end of the deformation process. Thus, all of the bent beams in the PXCM sample contribute to the energy dissipation. Despite this, the energy dissipation capacity of this PXCM is slightly lower (approximately 12% for *W*_v_ and 18% for *W*_m_) for the {45°, 135°} loading case than the {0°, 90°} loading case (see Tables S4–S6). This is also due to the lower energy dissipation associated with the transition of each bent beam when the *a*_1_ axis of the material is inclined to the applied displacement. The experimental *F-d* curves for three cycles are presented in Fig. S5(b).

Figure 4(c) also shows two somewhat different regions in the *F-d* responses. The material seems to respond differently in the applied displacement range of 0–90 mm than for displacements greater than 90 mm. In the experimental response, there is a lower force plateau that extends over displacement range of 0–90 mm and a higher force plateau that spans the rest of the displacement range. The simulated response shows a smaller difference in the plateau force levels across these two displacement ranges, but it shows more prominent serrations in the higher displacement range than in the lower one. We also observe that the phase nucleation regions and transformation fronts (see red colored beams in Fig. 4(b)) no longer neatly follow motif rows or columns. Instead, we see zig-zag patterns all the way until densification. Both of these observations suggest the existence of preferred phase transformation propagation bands. The lower energy transformation fronts seem to take the form of long wavelength triangular waves aligned with the edges of motifs. The higher energy fronts also have the shape of a triangular wave, but these have a shorter wavelength. In a sample of relatively small size, as is the case here, the bent beams along the lower energy transformation propagation paths get exhausted when the sample is compressed through a displacement equal to 90 mm (60% of maximum displacement), and the material changes over to the bent beams along the less preferred transformation propagation paths for higher displacements. The small differences between various beams in a fabricated sample lead to an elevated plateau force level for displacements greater than 90 mm in the experimental case. However, as all of the bent beams are nominally identical in the FE simulations, groups of these beams transform together. This gives rise to the larger oscillations in stress in the simulated response for displacement above 90 mm.

**Figure 5:**
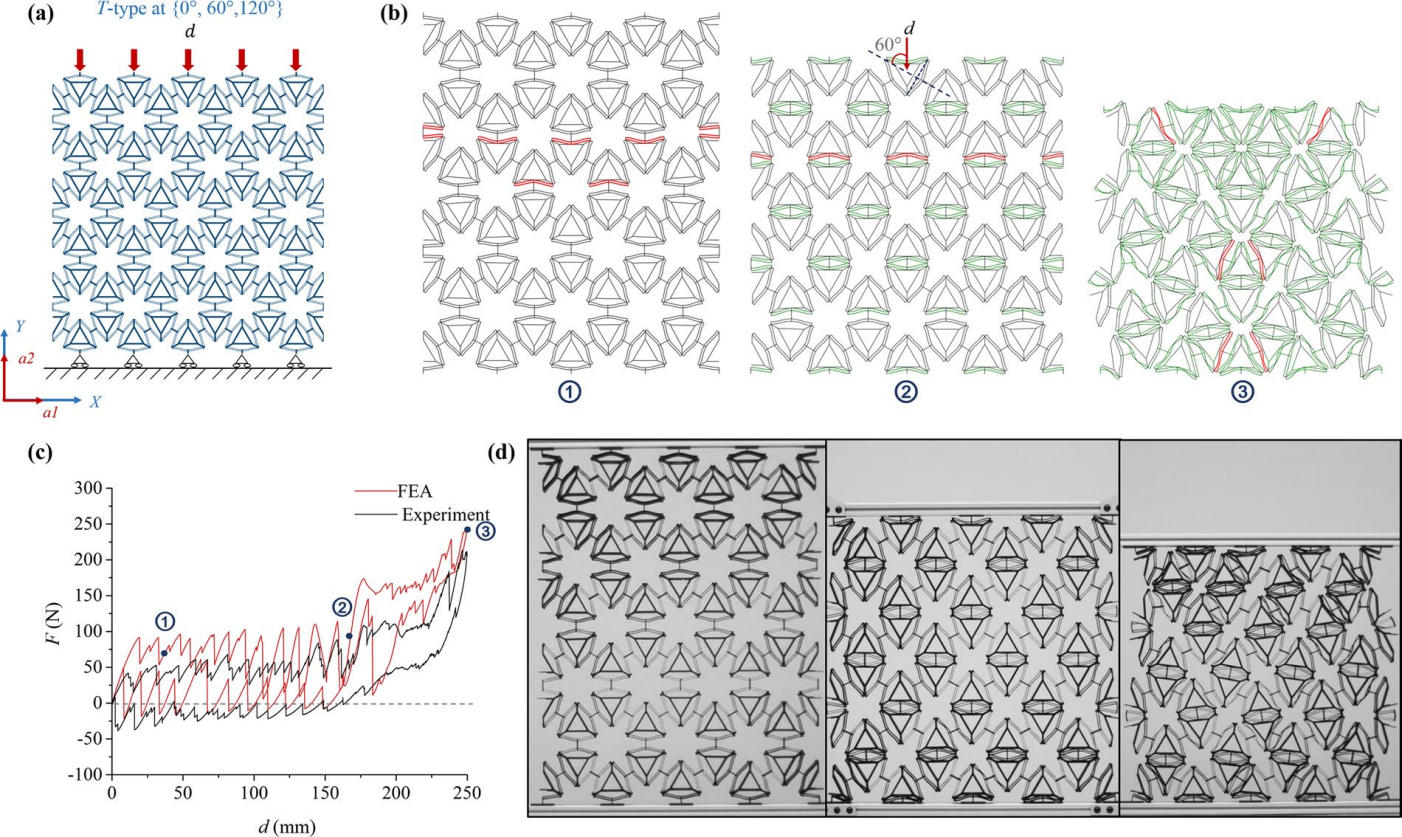
Performance of a *T-type* PXCM sample under one compressive load-unload cycle at {0°, 60°, 120°}. (**a**) The sample is under uniaxial loading condition and supported by rollers at bottom. (**b**) Phase transformation sequence of the three characteristic states from FE simulation. (**c**) *F-d* relation of sample from FE simulation and Experiment. (**d**) Three states of the sample from initial to final deformed configurations (See Video 3 in the supplementary material).

The mechanical response (see Fig. 5) of the *T-type* PXCM under uniaxial loads applied along {0°, 60°, 120°} shares features of the mechanical responses seen in the *S-type* PXCMs under both {0°, 90°} and {45°, 135°} loadings, but is more similar to the latter case. The simulated and experimental *F-d* responses are qualitatively similar. Both responses show some common features but the serrations in the experimental response are much less distinct than those in the simulated response. Both responses show two distinct regions. The experimental and simulated *F-d* responses show serrated loading and unloading plateaus over the displacement range 0–170 mm. There is a jump in the plateau force levels associated with both branches for displacements above 170 mm. The latter region is also characterized by barely discernable serrations. We observe that the material behaves like a metastable PXCM for displacement above 170 mm and as a bistable PXCM for lower displacements. The experimental *F-d* curves for three cycles are presented in Fig. S5(c).

As in the case of the PXCM with square shaped motifs when it is loaded along {45°, 135°} degrees, this dichotomy in the responses can be traced back to the inclination of the applied displacement to the ${a}_{1}\,$ axis of the material. Approximately a third of the bent beams in this case are orthogonal to the loading direction. These bent beams require lower transformation force and transform first and in an orderly progressive manner as suggested by the phase transformation front shown in Fig. 5(b). The loading direction makes a fairly steep angle (60°) with the axis of symmetry of the remaining two- thirds of the bent beams in the sample (Fig. 5(b)). This reduces the tendency of the mechanisms to snap-through, but they still need a sizable displacement component that is normal to line joining the ends of the beams to complete the forward transformation. This results in less distinct serrations as well as a higher plateau force for the transformations in these cells. The compression in simulation and experiment of this sample was stopped before it reached the theoretically determined maximum displacement because of the extreme distortion of the motifs that is seen in the last pane of Fig. 5(d).

**Figure 6:**
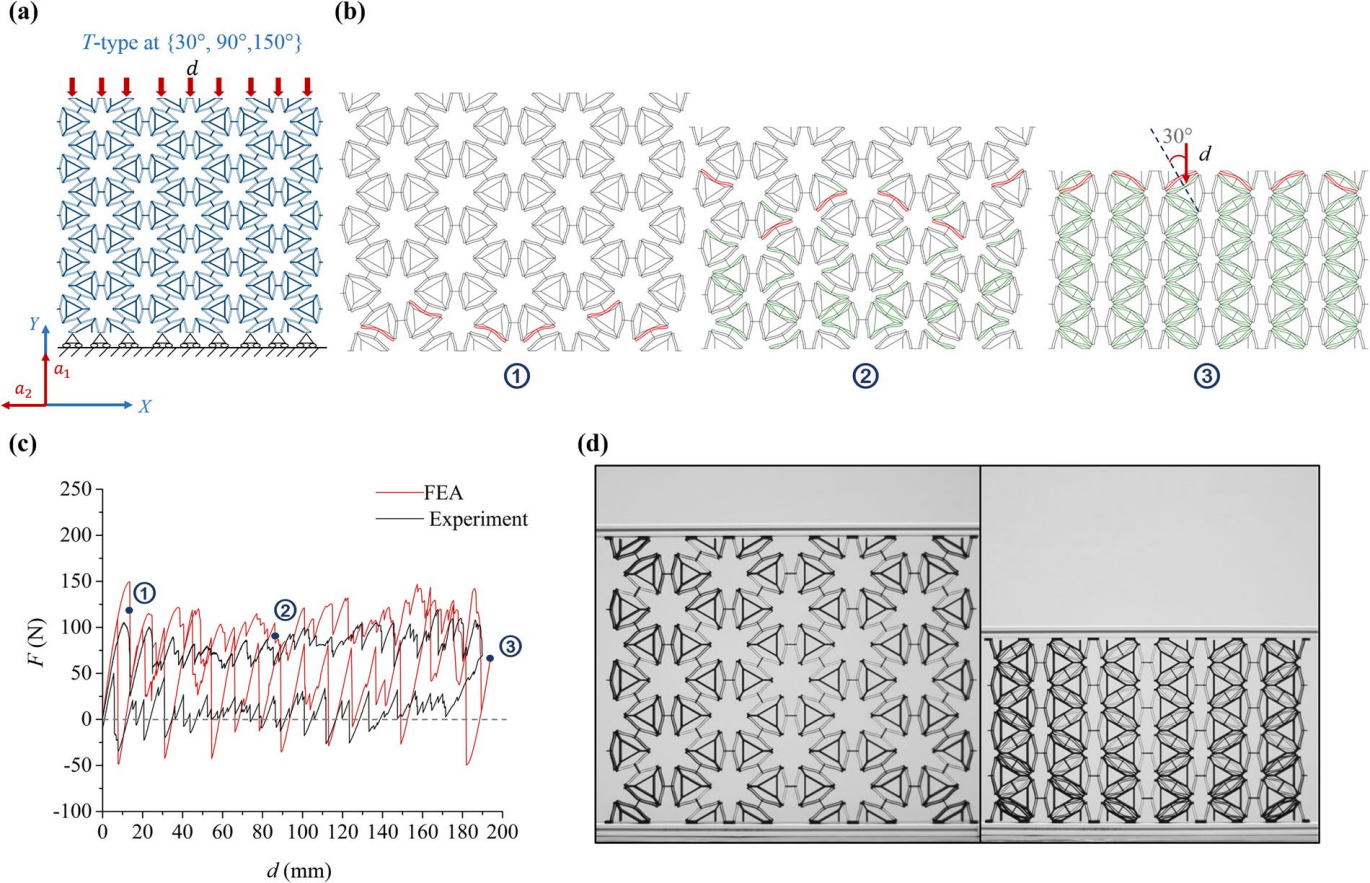
Performance of a *T-type* PXCM sample under one compressive load-unload cycle at {30°, 90°, 150°}. (**a**) The sample is under uniaxial loading condition and supported by rollers at bottom. (**b**) Phase transformation sequence of the three characteristic states from FE simulation. (**c**) *F-d* relation of sample from FE simulation and Experiment. (**d**) The states of the sample at initial and final deformed configurations (see Videos 4 and 5 in the supplementary material).

The mechanical response of the *T-type* PXCM under uniaxial loads applied along {30°, 90°, 150°} degrees (see Fig. 6(a)) shares features of the mechanical responses seen in the *S-type* PXCMs under both {0°, 90°} and {45°, 135°} loadings, but is more similar to the former loading case (shown in Fig. 6(b,c)). The *F-d* response in Fig. 6(c) shows qualitative agreement between the simulated and experimental responses. Both show serrated loading and unloading plateaus that are sufficiently separated for the material to exhibit energy dissipation. The serrations are not as regular or distinct as in the {0°, 90°} loading case for the *S-type* PXCM, but they are sufficiently regular and distinct to allow us to identify individual peaks in the loading (or unloading) branches with the forward (or reverse) transformation of one bent beam structure in a column of motifs. This can be explained by the larger inclination angle (30°) between the loading direction and axis of symmetry of two-thirds of the bent beam mechanisms in the sample (Fig. 6(b)). The phase transforming band fronts in Fig. 6(b) follow a slightly zig zag path that mostly hews to horizontal rows of motifs. Thus, we observe a mostly orderly progressive transformation of rows of bent beams until the sample is fully compressed. This is also reflected in the uniformity of the serrations in the *F-d* response.

The experimental response indicates a PXCM whose behavior is right at the boundary between bistable and metastable (Fig. 6(c)). The experimental *F-d* curves for three cycles are presented in Fig. S5(d). The material behaves mostly like a metastable PXCM during the compression of the sample (loading branch), but the sample retains the compacted configuration reached at the end of the loading branch without the need for an external force to hold that configuration. A small tensile force is needed to initiate and subsequently nudge the reverse transformation to completion. The simulated response is similar to the previous two loading cases showing more pronounced serrations in the unloading branch than those observed in the experiments. We also note that approximately two thirds of all bent beam mechanisms in this PXCM sample undergo phase transformation in this case (see Fig. 6(d)). The remaining mechanisms that are approximately parallel to loading direction remain in phase 1 at the end of the compression process (Fig. 6(b,d)). Recall that almost all the mechanisms in the *T-type* PXCM loaded along {0°, 60°, 120°} underwent phase transformation. As in the case of the *S-type* PXCM, we observe that, because there are more bent beams in a *T-type* PXCM that undergo phase transformation in for loading along {0°, 60°, 120°} than for loading along {30°, 90°, 150°}, the energy dissipation associated with each of the transitions is lower in the latter case. There results indicate that the *S-type* and *T-type* PXCMs are similar in terms of energy dissipation performance (approximately 12% difference for both load cases).

## Discussion

**Figure 7:**
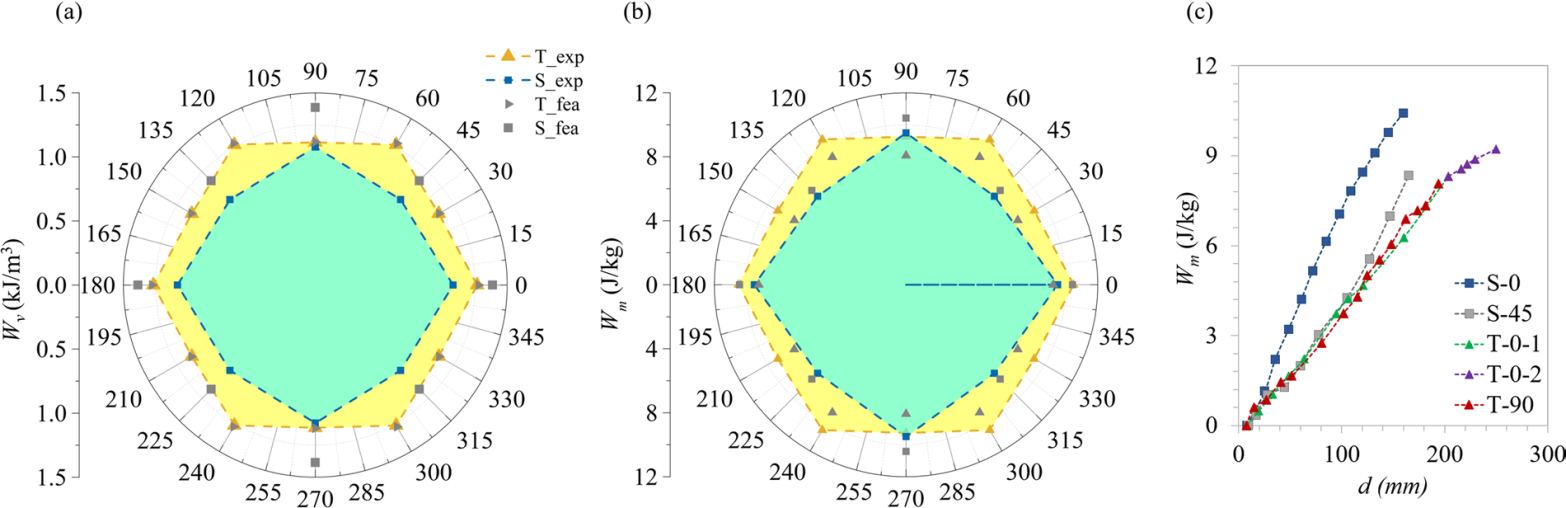
(**a**) *W*_*v*_ – energy dissipation per unit volume, (**b**) *W*_*m*_ – energy dissipation per unit mass, as a function of the loading angle for the two 2D PXCMs presented here. Gold triangular and blue square symbols represent experimental results of *T-type* and *S-type* PXCMs. Gray triangular and square symbols represent simulation results of *T-type* and *S-type* PXCMs. (**c**) Energy dissipation *W*_*m*_ varies with applied displacement.

The variation in the energy dissipation performance of the two 2D PXCMs with the axis of loading is an important functional attribute of the 2D PXCMs. In this section we compare the performance of the two 2D PXCM designs based on two metrics: *W*_*m*_ (energy dissipated per unit mass) and ${W}_{v}$ (energy dissipated per unit volume) for loads applied along various axes of symmetry of the materials. We also estimate the fraction of the total dissipated energy that is dissipated via pathways other than the snapping action of the beams in the samples. Panels (a) and (b) in Fig. 7 summarize respectively the volume-specific (${W}_{v}$) and mass-specific (${W}_{m}$) energy dissipation capacity as a function of the loading angle for the two 2D PXCM designs presented earlier. The average energy dissipated by a sample in cycles 2 and 3 in the experiments and the FE simulation can either be normalized by the volume of the undeformed bounding box that encloses the sample (see Fig. 2 and Table S2) or by the mass of the sample to get its ${W}_{v}$ and ${W}_{m}\,$ performance respectively. Energy dissipation in PXCMs occurs in a discontinuous way through discrete steps corresponding to snap through transitions in individual building blocks. Therefore, energy dissipation per unit displacement cannot be defined in the traditional way involving ratios of infinitesimal changes in energy dissipation and displacements. However, we can define an average energy dissipation rate in the following way: we load the material up to a snap through at a displacement *d*_1_, and then unload it completely. As such the average energy dissipation rate at *d*_1_ can be defined as the ratio between the energy dissipated in this complete load-unload cycle, *W*_*m*_ (*d*_1_) and the applied displacement *d*_1_. This is repeated for all snap through events to get the average energy dissipation, *W*_*m*_*(d*), for different displacement values through its loading history. Figure 7(c) shows *W*_*m*_ a function of *d* calculated from the FE models. With the exception of the *S-type* PXCM for {0°, 90°}, that exhibits a higher energy dissipation rate, the rest of samples show similar performance. More discussion can be found in Supplementary Materials Section S5 (Table S12).

A couple of observations are noteworthy: (1) ${W}_{v}$ and ${W}_{m}$ for both 2D PXCM designs do not vary significantly with the loading direction for loads applied along the various axes of symmetry for the materials. (2) The *T-type* PXCM has a small performance advantage over the *S-type* PXCM in terms of both - ${W}_{v}$ and ${W}_{m}$. These observations are not readily apparent when we recall (see Figs 3–6) that the mechanical responses of the two 2D PXCM designs were quite different under loads applied along the various axes of symmetry for the materials (shown in Fig. S6). Furthermore, those results do not shed any light on how the material might respond when it is subjected to loads that are applied along a more general direction in the plane i.e. not along an axis of symmetry for the material. We carried out ancillary analysis done on a single mechanism comprising two parallel bent beams to understand the role of the loading directions on its bistable behavior (see Section S3.2). We found that the ability of this mechanism to exhibit bistability decreases with the angle between the loading direction and the axis of symmetry of the bent beam to the extent that the mechanisms ceases to exhibit negative stiffness when the such angle is higher than 15° (see Fig. S7). In principle, this effect would degrade the capability of the material to dissipate energy. However, a closer examination at the PXCMs analyzed in Figs 4–6 reveals that the individual cells in a larger ensemble reorient themselves during the deformation of the material, such that they reduce the inclination of the force deforming a mechanism with respect to the axis of symmetry of the mechanism undergoing transformation improving their bistability and therefore their capability to energy dissipation (see Figs S8–10 in Section S3.2).

The variation in energy dissipation across successive cycles for all four load cases is less than 8% (Table S3*)*. The highest variations for the different load cases occur at the transition from cycle 1 to cycle 2 and are likely due to initial inelastic behavior of the sample and stress concertation. There is very little variation from cycle to cycle across subsequent cycles (Table S4). The overall highest variation is reported for the *S-type* PXCM sample with the square based motif when it is loaded at {0°, 90°}. In addition to the snapping action of the mechanisms, other energy dissipation pathways may also contribute to the observed energy dissipation in the experiments e.g. (a) plastic dissipation within the polymeric base material, (b) viscous dissipation within the base material, (c) frictional dissipation due to rubbing between the sample and its boundaries, (d) friction between the sample and the guards that limit out of plane deformation, (e) friction between adjacent beams that come into contact during the deformation of individual mechanisms in the sample, and (f). internal friction between the various layers that are deposited by the rapid prototyping process. We consider the energy dissipation due to the snapping action of the beams to be the primary dissipation pathway for PXCMs. The total energy dissipated by PXCM sample can be attributed to the primary and secondary pathways is estimated (see S3.3, Tables S7–8).

In conclusion, we study functionally 2D *S-* and *T-type* PXCMs that exhibit significant energy dissipation when they are loaded along multiple directions. Experiments on 3D printed prototypes, together with nonlinear finite element simulations, were employed to examine and understand the mechanical behavior of these materials. The experimental results show that the energy dissipation capacity of both PXCM designs did not vary substantially with the direction of loading. We also note that the results reported in this work are for samples with a relatively small number of motifs. Replicating these experiments for samples with a significantly larger number of motifs is critical for understanding the true material response. We also report a study on a *T-type* PXCM under biaxial loading condition in the supplementary material (Fig. S19, Table S13, and Video 6). The analysis of 2D PXCMs response to more complex load cases such as bi-axial, bending and indentation is the focus of ongoing work that will be reported in the future. However, these materials offer a myriad of opportunities for developing applications such as those mentioned in the introduction. Moreover, we also observed that both designs exhibited auxetic behavior. *S-type* samples exhibited a Poisson’s ratio in the range of −1 to 0, whereas *T-type* samples exhibited Poisson’s ratio in the range −1.1 to −0.29 (See Fig. S12). Having these auxetic behavior indicates that 2D PXCMs might have additional mechanical advantages, such as high indentation resistance, shear modulus, fracture toughness, and synclasticity^27,28^, which extend the application of 2D PXCMs into medical stent^29^, and self-adaptive attire^30^.

## Materials and Methods

A Fortus 450MC fused deposition modeling machine from Stratasys is used to fabricate the PXCM samples using an ABS-based proprietary material called ABS-M30 (*E* = 2.28 GPa, ${\sigma }_{y}$ = 45 MPa). The thickness of a bent beam is a critical dimension that drives the size of the sample. Very thin beams lead to small overall sample sizes but these designs are susceptible to significant variations in beam thicknesses across the sample. We choose *t* = 0.7 mm to balance the need to keep the overall sample size small with the desire to ensure fairly consistent beam thicknesses across the sample. Each sample is made by assembling multiple 3D printed parts, because the complete samples have a bigger footprint than the build envelope of the 3D printer. These parts are then adhesively bonded to create the test samples. The samples are conditioned at room temperature and humidity for at least 48 hours before testing them. Further examination revealed that the adhesive and assembly method does not affect the mechanical response of the specimen.

The samples are tested in uniaxial compression in a universal test machine (MTS Insight 10). Horizontal bars comprising cruciform Aluminum extrusions are used to secure the test samples in the test frame. The test samples are designed such that their top and bottom ends have ‘T’ shaped features that slide in the T-slots of the horizontal bars. The ends of the sample cannot move vertically nor can they rotate about any axes relative to the horizontal bars. A thin coating of a lubricant is applied to the slots to reduce friction between the samples and the horizontal bars, so that the sample ends can slide freely in the horizontal direction. The out-of-plane width of the sample is chosen to be sufficiently high (25 mm) to mitigate the tendency of the sample to buckle out of plane. However, as a precaution, diagonal bars spanning the entire sample (see Fig. S4(a)) are mounted behind the sample to brace it against deforming out of the plane of the sample. The edges of the samples are free from any boundary conditions.

Four samples that represent the two 2D PXCMs designs, each in two different material orientations, are compressed under displacement control at the quasi-static rate of 1 mm/min. The displacement is applied to ensure maximum amount of the mechanisms collapse (see Fig. S4(c)). The total force acting on the sample is measured by a load cell (MTS 661.19F-02, 10 kN capacity) and the cross-head travel is recorded as the displacement of the top end of the sample. Each sample is subjected to three back-to-back load-unload cycles to check for repeatability and any evidence of irreversible deformation (see Fig. S5). The area enclosed by the loading and unloading branches of the *F-d* response is the energy dissipated by the sample in that cycle. The average energy dissipation from the second and third cycles is used to represent the energy dissipation capacity of the material for that load case.

Although the PXCM samples are loaded in displacement controlled at a quasi-static global deformation rate of 1 mm/min, the limit point traversals in the various beams are dynamic events with fairly short characteristic times (Q(1 ms)). Moreover, contact between adjacent structural elements in the PXCM and the non-equilibrium jumps associated with the snap-throughs introduce significant nonlinearity in the response of the PXCM. Displacement controlled, direct integration, implicit dynamic finite element analyses in a finite element analysis software Abaqus 6.15 provides a robust way to simulate the complex behavior of these materials. The FE simulations in this work use a two node, shear flexible beam element discretization of the PXCM samples. Results from beam, 2D and 3D solid element discretizations were compared for accuracy of the solution as well as its computational cost (Fig. S13). The element sizes were determined through a convergence study (Fig. S14).

The mechanical properties employed in the FE simulations are those corresponding to ABS-M30 (which are reported in Section S4.4, Tables S9 and S10). Considering the experimental and material variability (i.e., lower and upper bound of these properties) the simulated *F-d* curves are reported in Fig. S17. In order to properly simulate the experimental boundary conditions shown in Figs 3–6(a), the corresponding FE simulations had a frictionless roller boundary at the interface with the ground, the sides were free from any displacement constraints or tractions and a uniform velocity of 1 mm/min was applied to all nodes on the top edge of the sample. Contact between adjacent beams was modeled using the small sliding formulation in Abaqus. Coulomb friction with a friction coefficient of $\mu =0.1$ was assumed to be active at all contact interfaces (See discussion in S4.3 and Fig. S15).

## Supplementary information

Supplementary Material
