# Supplementary material for: Energy dissipation in functionally two-dimensional phase transforming cellular materials
Source: Sci Rep. 2019 Aug 29;9:12581. doi: 10.1038/s41598-019-48581-8 (PMC6715794; doi:10.1038/s41598-019-48581-8)
Supplement: Supplementary file 1 — Supplementary Material [file 41598_2019_48581_MOESM1_ESM.pdf]

Supplementary materials for

Energy dissipation in functional two-dimensional phase transforming cellular materials

Yunlan Zhang, David Restrepo, Mirian Velay-Lizancos, Nilesh D. Mankame, Pablo D. Zavattieri

Corresponding author
Email address: zavattie@purdue.edu (P. Zavattieri)

S1. Design of 2D PXCMs

In this paper, we present a systematic study to design and study the mechanical performance of functional two-dimensional phase transforming cellular materials (PXCMs) that are capable of dissipating energy along various axes of symmetry. We created a series of designs and then we evaluated them through FE simulations (Fig. S1 and S2). While the extension to 2D PXCMs may seem easy by follow the schematics shown in Fig. 1, the process requires a careful examination of the performance of the different types of designs. As mentioned in section 2.2, 2D PXCMs have three levels of hierarchy structures from level zero to level two. The *zeroth* level of the hierarchy structure is the elementary building block of the PXCMs which are composed of either single (Fig. S1(a)) or a pair of parallel-connected bent beams (Fig. S1(b)). To evaluate both designs, the FE simulations of two *S-type* PXCMs samples with these two types of building blocks under a uniaxial load-unload cycle are created.

We observe that the sample with single bent beams as elementary building blocks shows local wobbling behavior (Fig. S1(a)), which causes unpredictable and disorderly transformation behavior of materials. This local "wobble" mode is caused by the rotation at the apex of the single bent beam mechanisms. On the other hand, when the elementary building blocks are parallel-connected bent beams, materials transform steadily and progressively (Fig. S1(b)). This is mainly caused by the fact that rotation at the apex is restricted with parallel beams.

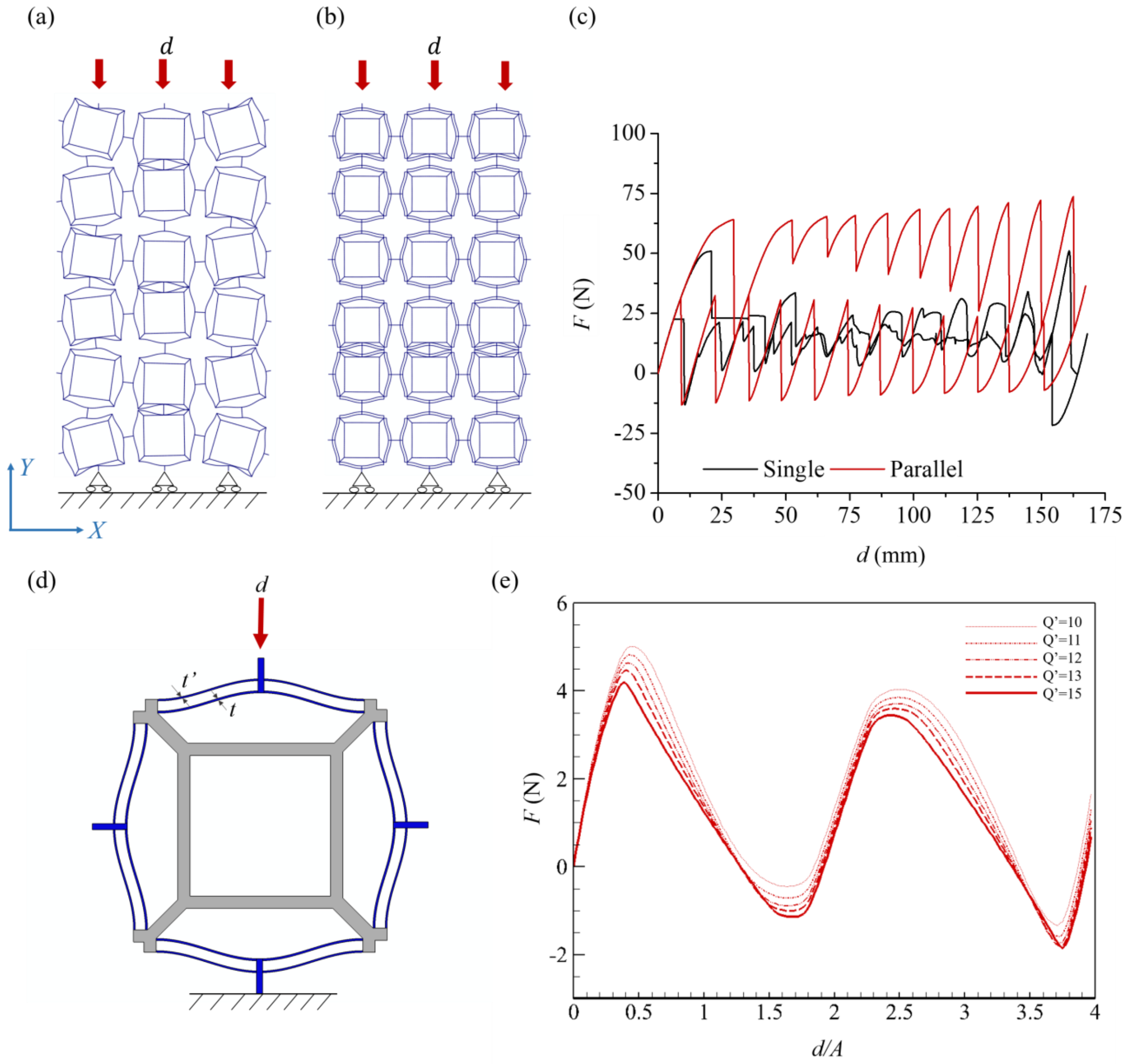

Figure S1: Performance of *S-type* 2D PXCMs with the zeroth level hierarchy structure are comprised of (a) single and (b) parallel connected sinusoidal beams under a compressive load-unload cycle. (a) Unit cells with single bent beam exhibited strong rotation. (b) Unit cells with parallel-connected sinusoidal beams did not rotate. (c) *F*-*d* relation of two samples indicates that *S-type* 2D PXCM with parallel-connected bent beams as elementary building blocks dissipates more energy. (d) *S-type* 2D PXCM motif under a uniaxial load. (e) Motif exhibits higher bistability but lower peak and valley forces when the top bent beam thinner.

Figure.S1(c) shows the result of the simulations (Load-Displacement, *F*-*d* curves) of the both cases (single beam and pair of parallel-connected beams). These results indicate that materials with

parallel-connected bent beams as *zeroth* level of the hierarchy structure can have higher better bistability behavior (e.g., larger peak to valley force ratios) which leads to better energy dissipation capacity. Additionally, the thickness of each individual beam in the two parallel-connected bent beams can be tailored independently to influence the performance of the material. To understand the effect of having different values of thickness in the top and the bottom bent beams, we performed FE simulations of a group of *S-type* 2D PXCMs under the displacement-controlled uniaxial compression. For each building block, the amplitude A and wavelength λ of both bent beams, the thickness t of the bottom bent beam, and supports thickness $t_{stiffer}$ are identical among the simulations. The only variation among different simulations is the thickness t' of the top bent beam (Fig. S1(d)). We define a parameter $Q' = A/t'$ as an indicator of the value t' *(since we kept A constant)*. The parameter Q' varies from 10 to 15 among our simulations (Fig. S1(e)). The rest of the dimensions follow those indicated in Table S1. The relation between force and normalized displacement *(d/A)* of all the simulations are displayed in Fig. S1(e). These *F-d* curves indicate that, when all the other geometry parameters remain constant, the thinner the top bent beam is, the lower the peak and valley forces are, and the mechanism is said to become "more bistable". This means that the bottom part of the force-displacement curve cross the $F = 0$ line (e.g., the valleys remain negative while the peaks are positive), and the distance between peaks and valleys increases. Another interesting observation is that, during phase transformation, the top bent beam transforms first because the only lateral constraints are provided by the vertical stiffening walls. However, these top beams push the stiffening walls apart, reducing the constraints on the bottom bent beam. This competing mechanism can be tailored with additional analysis by finding the right combination of Q' and Q. However, to maintain the focus of the paper, most of PXCMs considered in this work have parallel bent beams with the same in thickness ($Q=Q'=10$, $t=t'$; see Table S1).

After analyzing these two design options at *zeroth* level of hierarchy structure, we studied two potential design options in the *first level of* the hierarchy structure of 2D PXCMs. Triangular and square motifs are natural candidates for building the *first* hieratical level of 2D PXCMs because they can be tessellated in a 2D plane. The building blocks are assembled by combining the bent beams together into *first* level motif. The first type of frame connects the bent beams from their ends with spokes that converge at the center of a motif (Fig. 1(d)). The second type also connects all the bent beams via the ends, but form a polygon frame inside a motif. To evaluate both design choices, we perform FE simulations of *S-type* 2D PXCMs with these two types of frames as it is shown in Fig. S2(a) and Fig. S2(b). Under a displacement-controlled compressive load-unload cycle, both specimens collapsed steadily. However, the *F-d* curves (Fig. S2(c)) indicate that the specimen with spokes (Fig. S2(a)) exhibits almost no hysteresis compared with the specimen with the center frame (Fig. S2(b)). Therefore, with adopt the center frame as the choice for *first* level of hierarchy (see Fig. 1(d)).

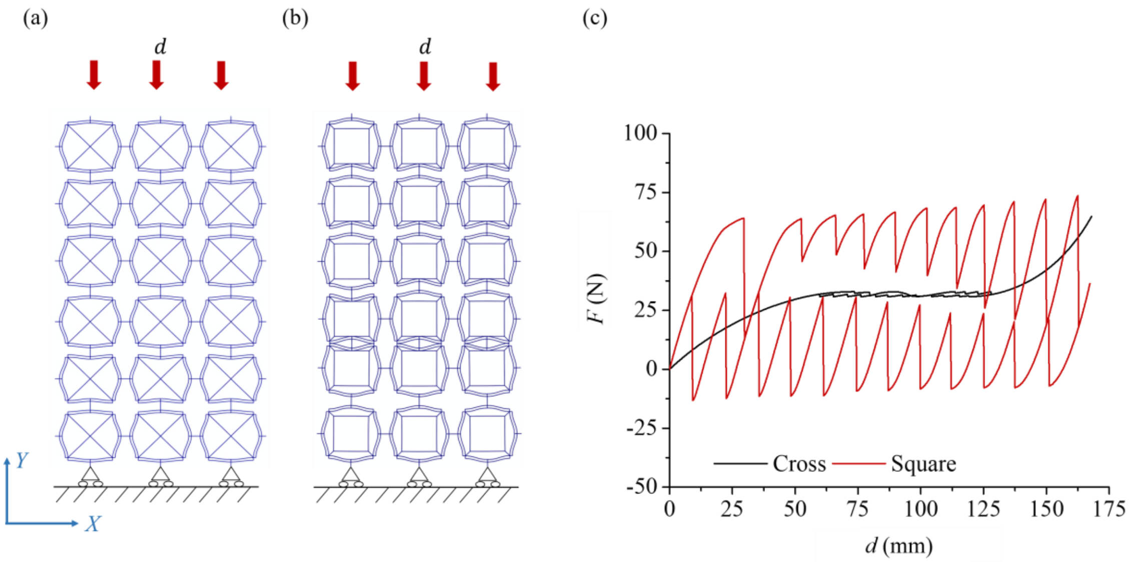

Figure S2: Performance of *S-type* 2D PXCMs with the two types of frames inside their motifs under a compressive load-unload cycle. (a) FEA model of *S-type* 2D PXCM with cross structures inside their motifs. (b) FEA model of square 2D PXCM with polygon frames inside their motifs. (c) *F-d* curves of two designs indicate that with polygon frame inside a motif, *S-type* 2D PXCM can dissipate more energy.

The determination of the second level of hierarchy follows a similar analysis through FEM analysis. The square motifs can be tiled easily as shown in Fig. 1(e). Choosing the support structures for these triangular shape motifs is not straightforward. Two alternative support structure topologies T_I and T_{II} are shown in Fig. 1(e). To evaluate the support structure topology T_I, a corresponding prototype is fabricated by an Object Connex500 3D printer with a photo-cured polymer (RGD 8530, $E = 1GPa$ and $\sigma_y = 19\ MPa$). The bent beams are designed to remain elastic during the phase transformation [1,2]. Ten compressive load-unload cycles were applied on the specimen using a universal testing machine (MTS Insight 10 equipped with a 10 kN load cell MTS 661.19F-02) (Fig. S3(a) and Fig. S3(b)). We observe that the supports and frames of the PXCM deform just enough to eliminate any constrain to the bent beams to obtain bistability. The hysteresis curves indicate that the energy dissipation is mostly plastic deformation rather than phase transformation. Therefore, regular tiling (T_I) does not lead to a functionally two-dimensional PXCM. The arrangement shown in (T_{II}) with triangular motifs located at the nodes of a regular hexagon is not a tiling as it includes some empty space at the center of the hexagon. As it is shown in the main paper, this arrangement leads to a functionally 2D PXCM. Optimization could be performed to improve the performance of 2D PXCMs in terms of energy dissipation, strength, or initial stiffness.

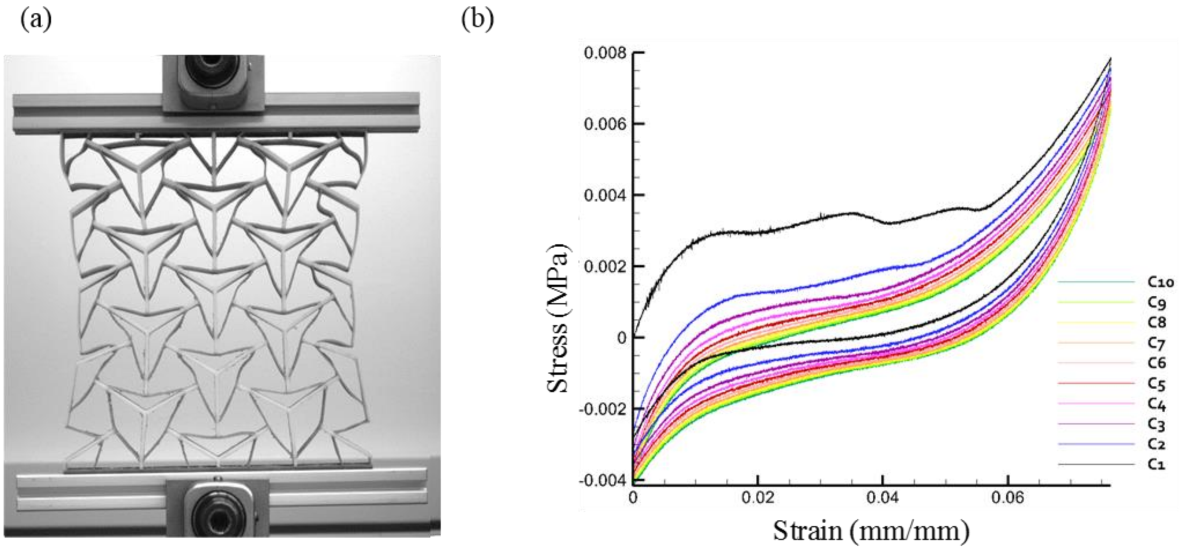

Figure S3: Example of an unsuccessful design. *T-type* 2D PXCM with T_I arrangement show plastic deformation under ten compressive load-unload cycles.

S2. Characterization 2D PXCMs

Uniaxial, quasi-static, compressive load-unload tests were performed to characterize the response of the *S-type* and *T-type* PXCMs along the various axes of symmetry of the materials. These tests were carried out under displacement control. Four specimens corresponding to four tests are fabricated (Fig.3-6). The volumes of samples are displayed in Table S2. These load cases are tested using nonlinear finite element analysis. This analysis helps us understand whether or not there is phase transformation, acknowledging that the applied displacement in the beams is a function of θ, where θ is the angle between the loading direction and axis of symmetry of a bent beam (see Fig. 4(c)). The applied displacement on each sample follows Eq. *s1*:

$$d = 2A \sum_{i=1}^{n} cos(\theta_i) \qquad (s1)$$

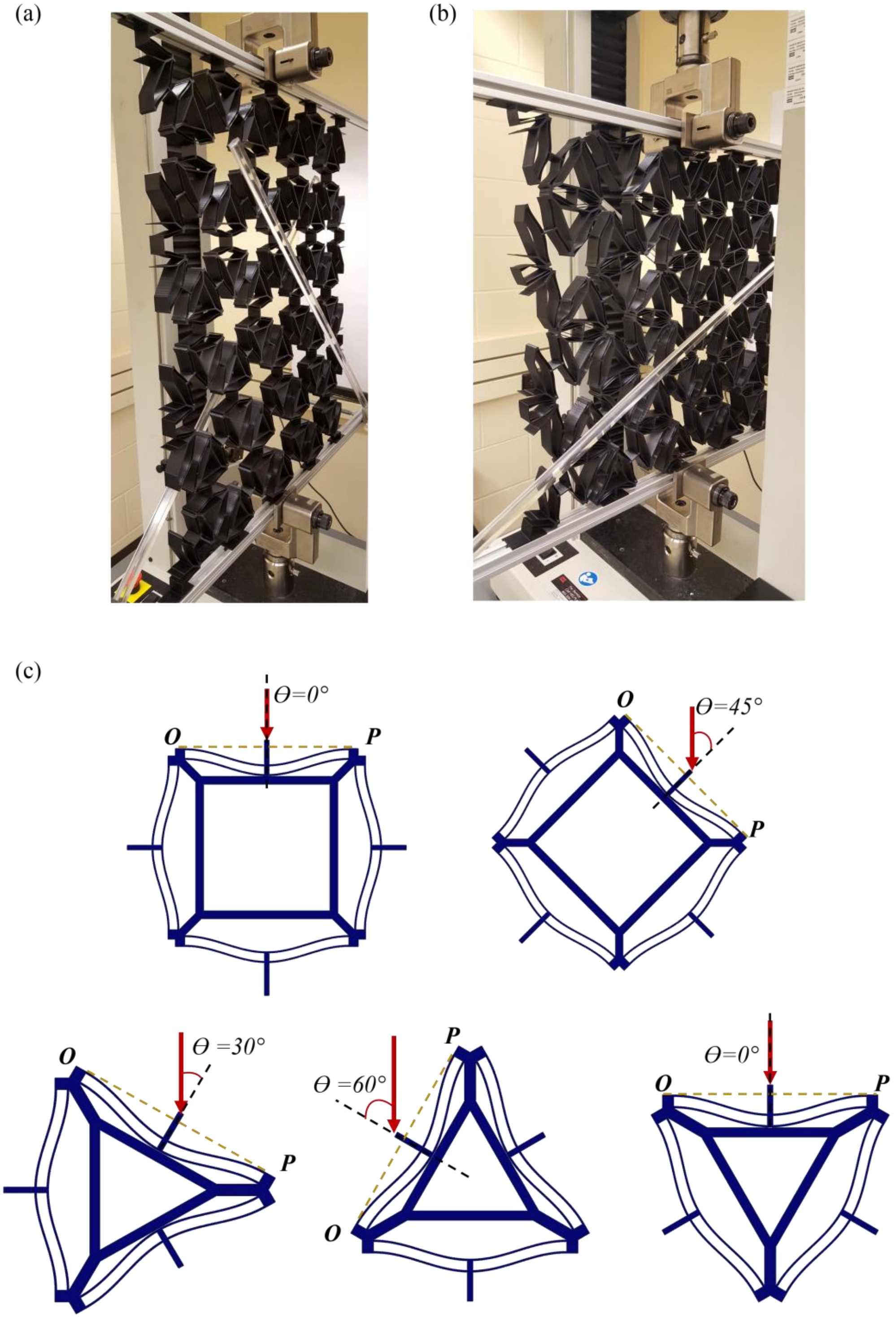

Figure S4: Experimental set up for the uniaxial loading tests on four samples. (a) undeformed and (b) deformed T-type at loading {0°, 60°, 120°} under uniaxial loading condition. Two acrylic bars are employed to laterally constrain the specimen at front and back to eliminate out of plane buckling. (c) FEM results showing how the cells undergo complete phase transformation, even when the applied displacement is a function of the angle θ.

where A is the amplitude of a bent beam, θ_i is the angle between the axis of symmetry of the ith bent beam structure and the loading direction, and n is the total number of such bent beam structures along any column of motifs in the material sample. All the specimens are tested with three compressive load-unload cycles with the loading rate of 1 *mm/min*.

S3. Results and Discussions

Figure S5 shows the *F-d* curves of each sample under three compressive load-unload cycles from experiments. Table S3 displays the energy dissipation of all the specimens among three back-to-back load-unload cycle. The variation among different cycles is between 1% and 8%. With the exception of the *S-type* 2D PXCMs loaded at {45°, 135°}, all the samples exhibit energy dissipation reduction after the first loading cycle. After the first loading cycle, the energy dissipation variation between second and third cycles decreases (Table S3-S4).

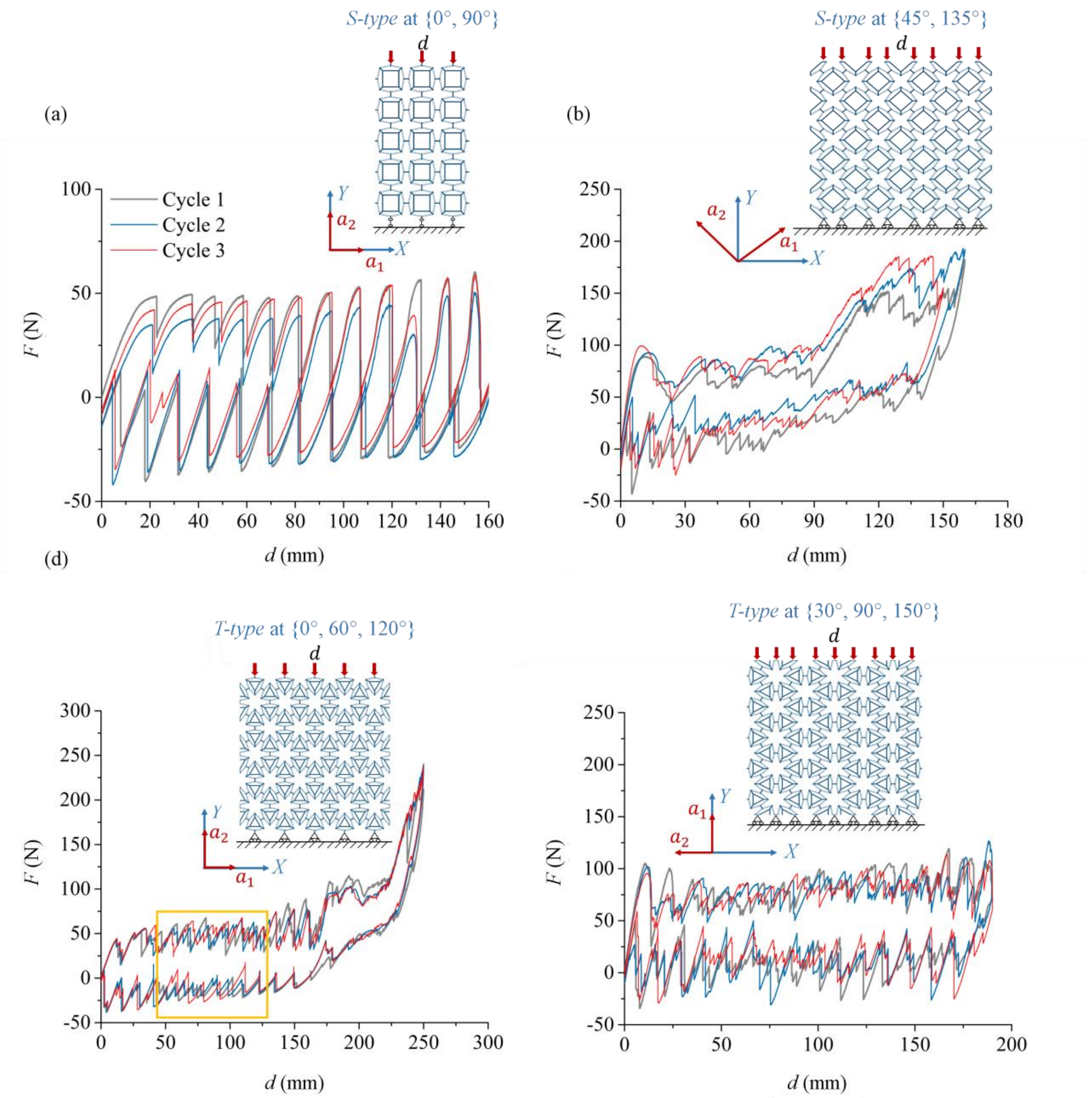

Figure S5: Experimental *F*-*d* curves for three compressive load-unload cycles. (a) A *S-type* PXCM sample under three compressive load-unload cycle at {0°, 90°}. (b) A *S-type* PXCM sample under three compressive load-unload cycle at {45°, 135°}. (c) A *T-type* PXCM sample under three compressive load-unload cycle at {0°, 60°, 120°}. (d) A *T-type* PXCM sample under three compressive load-unload cycle at {30°, 90°, 150°}.

S3.1 Phase transformation pathways

The observation from the tests confirm that most of the mechanisms under phase transformation through different level of asymmetry configurations. For instance, when the *S-type* 2D PXCM sample is loaded at {0°, 90°}, half of the bent beams happen to have their axes of symmetry overlapped with the loading direction. These bent beams transformed through the symmetric configurations, which is defined as the mechanisms go through the primary pathway (Fig. S6(a)). The other half of the bent beams do not go through phase transformation because the loading direction is perpendicular to their axes of symmetry. The *F-d* relation (Fig. S5(a)) of this specimen indicates that *S-type* 2D PXCM under loading angle {0°, 90°} show bistable behavior and energy dissipation. The materials perform differently when the loading direction for the sample is not aligned with axes of symmetry for a subset of the constituent mechanisms (Fig. S5(b)-(d)). When *S-type* 2D PXCM is loaded at {45°, 135°} loading angles (Fig. S5(b)), every bent beam has its axis of symmetry 45° rotated from the loading direction (θ=45°). This larger angle θ causes all the bent beams transform though more asymmetric configurations (Fig. S6(b)) compared with the previous case. As a result, the specimen exhibits mostly metastable behavior and lower energy dissipation capacity. In such cases, when a mechanism transforms via such an asymmetric configuration, we say that those mechanisms go through a secondary pathway. When the *T-type* 2D PXCM is loaded at {0°, 60°, 120°}, one third of bent beams with their axes of symmetry align with the loading direction. These bent beams go through the primary pathway; similar to half of the bent beams in the *S-type* 2D PXCM at loading angles {0°, 90°}. The rest of bent beams, which have their axes of symmetry 60° rotated from the loading direction, transform through the asymmetric configurations and exhibit metastable behavior (Fig. S6(c)). The *T-type* 2D PXCM at {30°, 90°, 150°} has one third of bent beams parallel to the loading direction. These bent beams clearly do

not exhibit phase transformation. The rest of bent beams have their axes of symmetry 30° rotated from the loading direction (θ=30°). These bent beams transform through the noticeable asymmetric configurations, therefore we define the mechanisms go through secondary pathway as well. Since the angle θ is relative small compared with the previous two cases, the sample shows bistability and relatively high energy dissipation (Figure S6(d)).

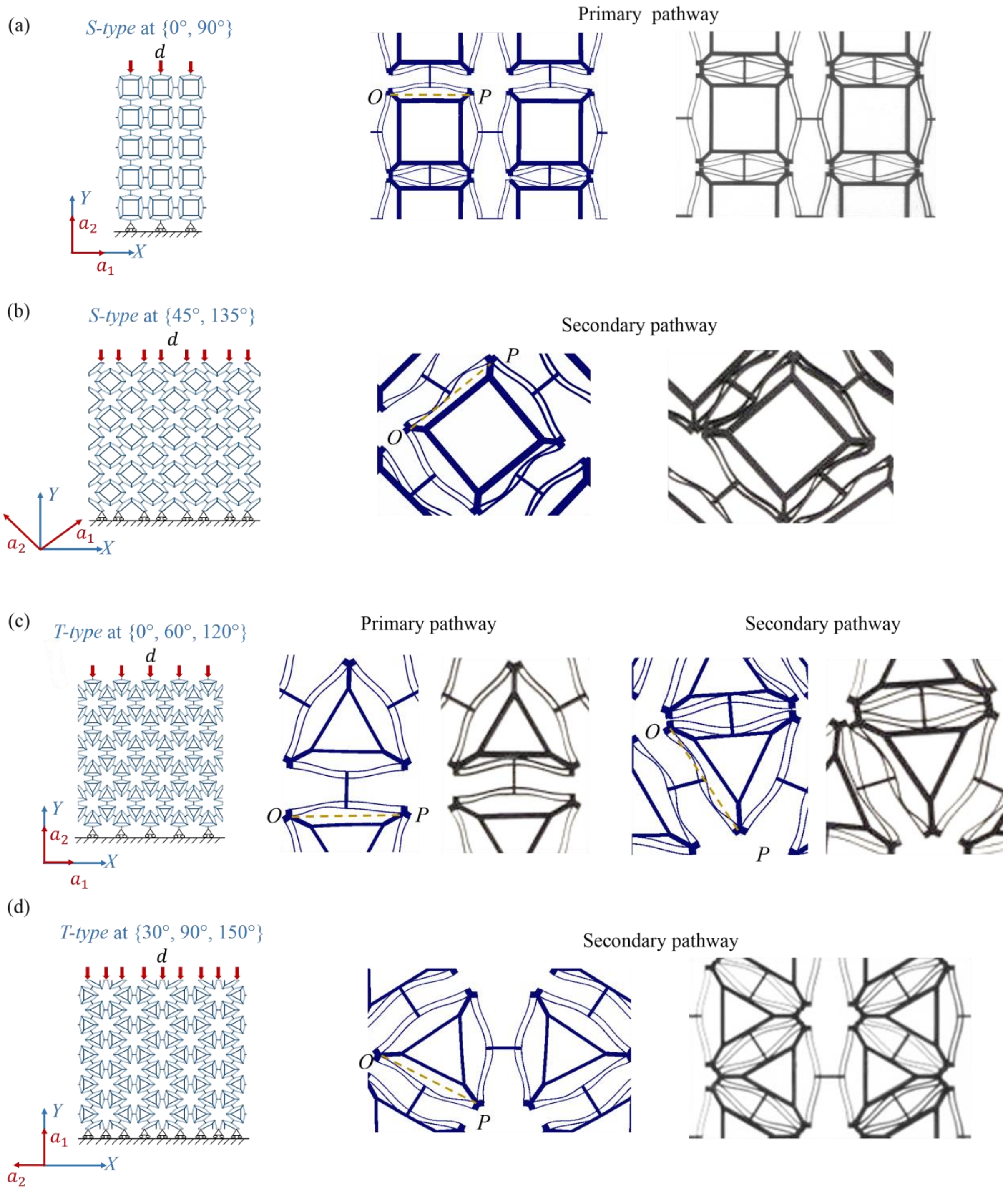

Figure S6: The simulations (left) and experiments (right) show that the bent beams have phase transformation through more asymmetry configurations when the angle θ between the loading direction and their axes of symmetry increase.

S3.2. Phase Transformation in a Single Mechanism

The experiments and FE simulations discussed in this paper show that the angles between loading directions and axes of symmetry of bent beams affect the performance of materials in terms of bistability and energy dissipation capacity. In order to better understand this influence, we perform ancillary FE simulations of a single mechanism under loads applied at various angles, θ (see Fig. S7(a)). Figure S7(b) shows the *F*-*d* responses as a function of the angle θ. We note that the mechanism response is clearly bistable for θ =0 with a well defined second stable configuration and a long region with negative stiffness. However, the mechanisms becomes metastable as θ increases, and the negative stiffness region in the response shrinks rapidly as θ increases from 0° to 15°. The negative stiffness region disappears completely for θ > 15° (see Fig. S7(b)).

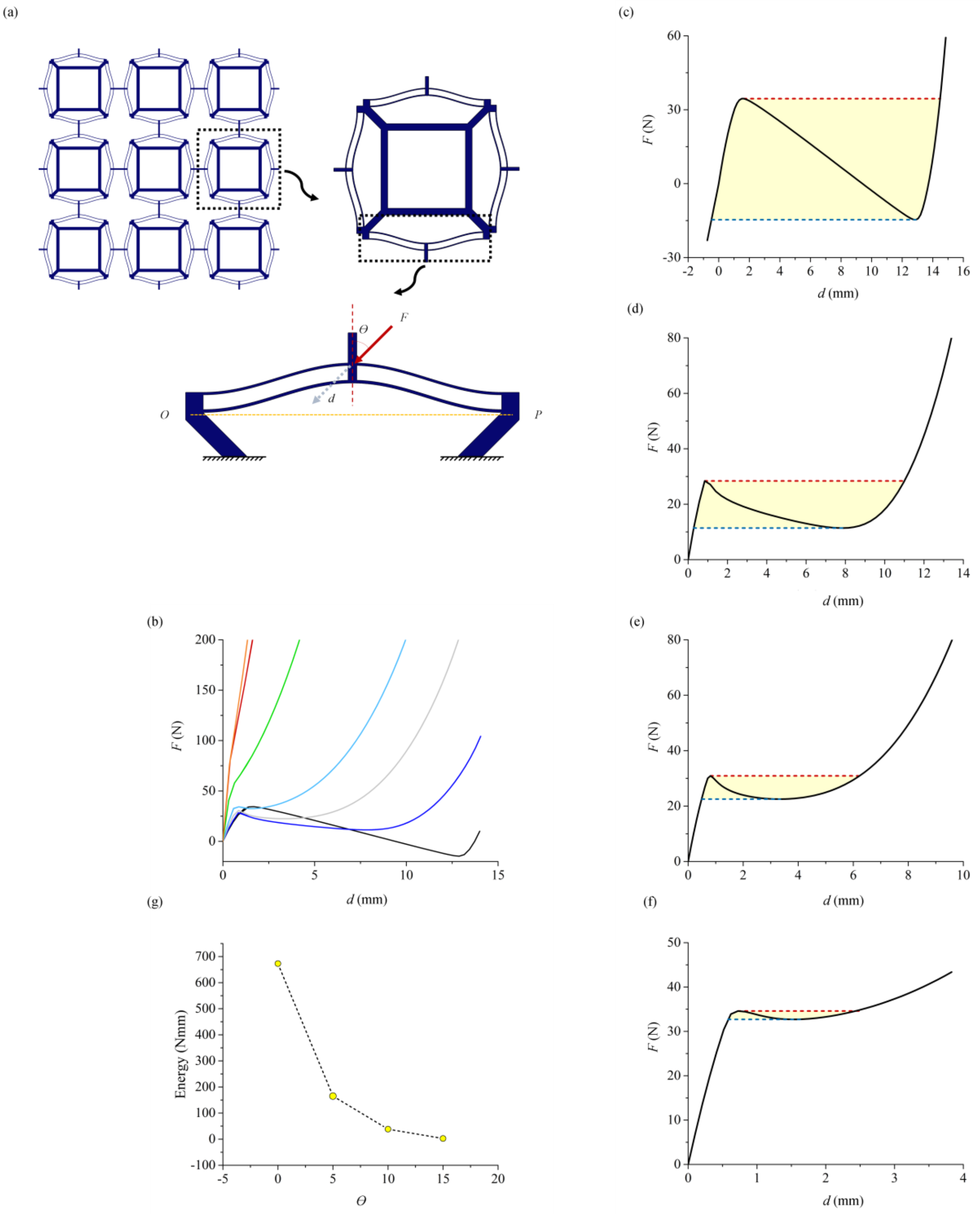

Figure S7: Performance of a single mechanism under the different loading direction. (a) A mechanism from *S-type* 2D PXCM is subtracted to study. (b) *F-d* relations of the mechanism when the angle between loading direction and axes of symmetry increases from 0° to 60°. (c)-(f) *F-d* relations of the mechanism when θ=0°,5°,10°, and 15°. Highlight area is proportional to the energy dissipation in materials is composed of a large number of mechanisms. (g) This area decreases exponentially with increasing θ.

As discussed, energy dissipation in PXCMs arises from the non-equilibrium release of energy accompanying the traversal of the limit points during the loading and unloading of these materials. Specifically, the energy dissipation in a material with a 'sufficiently' large number of motifs is proportional to the area bounded by the envelope curve shown by a dashed line in Fig S 7(c)-(f) [3,4]. Figure S7(g) shows a plot of this area as a function of θ. We note that the area appears to decrease exponentially with increasing θ and becomes nearly zero for θ =15°. This shows that the energy dissipation capacity of the single mechanism degrades quickly as the inclination of the applied load with respect to its axis of symmetry increases, and it disappears completely for inclinations as small as 15 degrees. Based on the observations, we conclude that the larger the angle between loading direction and the axes of symmetry of a bent beam, the lower their ability to produce bistable behavior. This eventually adversely affects the capability of the material to dissipate energy. However, the observations from the tests on 2D PXCMs show that when the angle θ is larger than 15°, the material still exhibit bistable behavior and energy dissipation capacity (Fig. S5). This is due to the collective behavior of all the cells, which produce enough lateral constraint even for those bent beams that have higher values of θ. This is discussed in the next subsection.

S3.3. Phase Transformation in a 2D PXCMs

The *S-type* 2D PXCM has four axes of symmetry, but only two of these are aligned with axes of symmetry for its constituent single mechanisms. When a loading direction for these PXCMs is aligned with an axis of symmetry of the material that also happens to be an axis of symmetry for a subset of its constituent mechanisms, this subset of mechanisms contributes the most to the total energy dissipation of the specimen. Half of the mechanisms in the *S-type* PXCM are aligned with their axis of symmetry lying along the 0 degree direction, and the other half have their axis of

symmetry along the 90 degree direction (see Fig. 3). The mechanisms aligned at 0 degrees are not deformed significantly, and hence do not contribute to the total energy dissipation of the sample when the mechanism is loaded along 90 degrees. Similarly, the mechanisms aligned with 90 degrees do not contribute to energy dissipation when the sample is loaded along 0 degrees. Moreover, as the same number of mechanisms are active contributors to the total energy dissipation when the material sample is loaded along 0 and 90 degrees, we expect the total energy dissipation to be similar in these two cases.

The situation is different when the loading direction for the sample is not aligned with axes of symmetry for a subset of the constituent mechanisms (Fig. 4-6). Based on the prior discussion, we expect any mechanisms oriented in a direction such that their axis of symmetry is inclined 15 degrees or more with respect to the load direction to have a very small contribution to the overall energy dissipation (see Fig. S7). However, an ensemble of mechanisms behaves somewhat differently than the single mechanism due to the internal degrees of freedom possessed by the individual motifs and its interaction with their neighbors. We notice that the individual cells in an ensemble reorient themselves during the deformation of the material such that they reduce the inclination of the force deforming a mechanism with respect to the axis of symmetry of the mechanism undergoing transformation (see Fig. S8-10). This reorientation happens via rotation of the individual motifs based on which subset of its constituent mechanisms is transforming. Thus, we can observe the same motif rotating clockwise and counter-clockwise at different points in the loading history (Fig. S9-10). This behavior results in some energy dissipation contribution even from mechanisms that were oriented such that the direction of their axis of symmetry was inclined by 15 degrees or more with respect to the external force in the undeformed configuration.

Moreover, the percentage of bent beams that undergo transformation in *S-type* 2D PXCM significantly increases from 50%, when it is loaded at 0°/ 90°, to 100% when a is loaded at 45°.

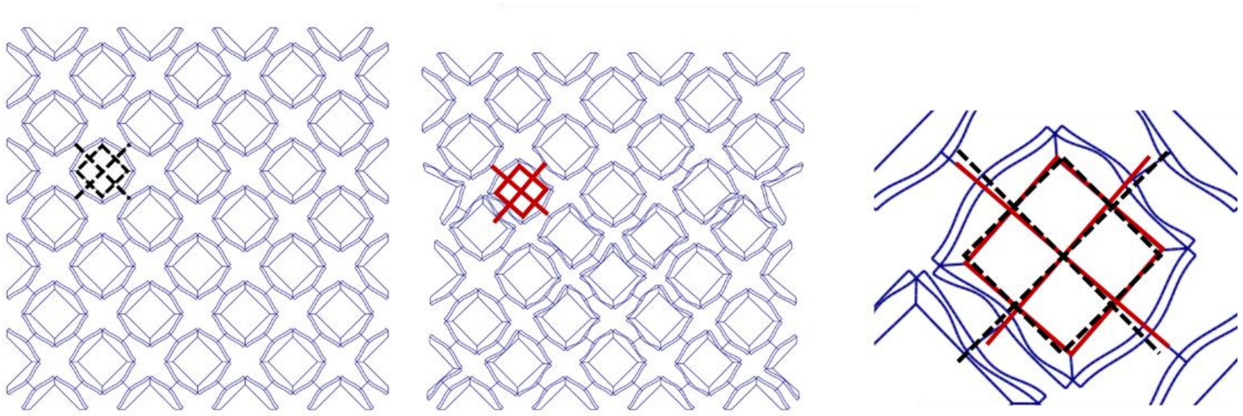

Figure S8: *S-type* 2D PXCM at {45°, 135°} loading angle show the rotation in the individual motif.

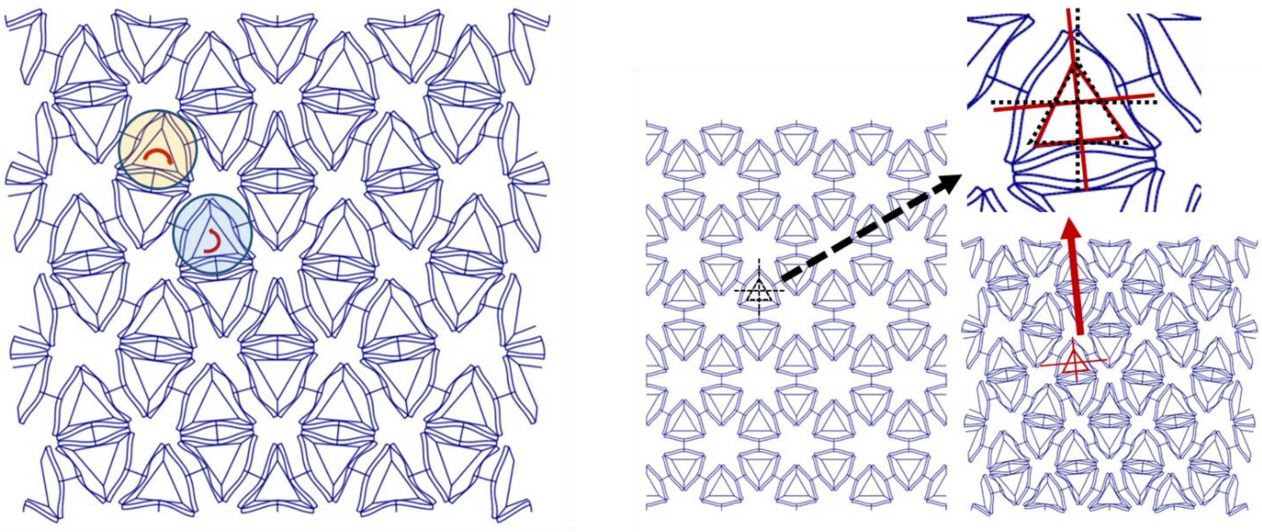

Figure S9: For *T-type* 2D PXCM at {0°, 60°, 120°} loading angle, the same motif rotating clockwise and counter-clockwise at different points in the loading history

Thus the relative small difference of the energy dissipation behavior of the *S-type* 2D PXCM to the direction of the in-plane loading can be explained by two counteracting trends: 1) a decrease in the energy dissipation contribution of an individual mechanism with an increase in the inclination of the applied load with respect to its axis of symmetry and 2) an increase in the number

of mechanisms contributing to the energy dissipation of the sample as a whole with an increase in the inclination of the applied load with respect to the axis of symmetry of any one subset of mechanisms. The behavior of both *T-type* 2D PXCMs can be explained in a similar manner.

We observe that the *T-type* material, which has more axes of reflectional symmetry then the *S-type*, exhibits slightly lower variation in energy dissipation with changes in the loading direction than the *S-type* material. This suggests that further increases in the number of axes of reflectional symmetry for the unit cell are likely to reduce the variation in energy dissipation with the changes in loading direction, with the advantage that the relative density of the material does not change significantly while increasing the symmetry of the unit cell.

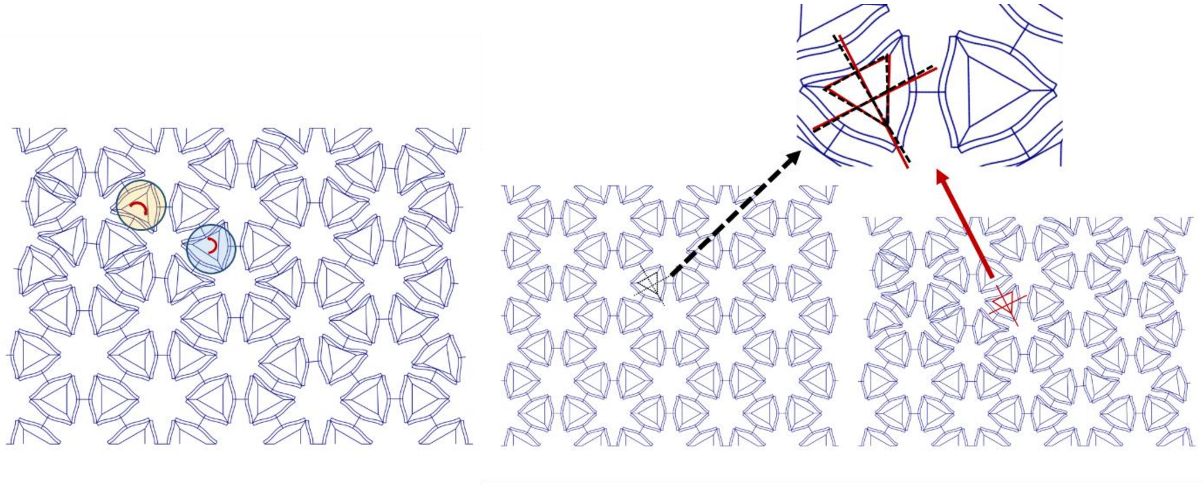

Figure S10: For *T-type* 2D PXCM at {30°, 90°, 150°}, the same motif rotating clockwise and counter-clockwise at different points in the loading history.

To verify the energy dissipation presented above is still produced by the PXCM when its base material remains in the elastic regime, we carefully analyze other type of sources for potential energy dissipation sources. This will allow us to examine and estimate how much of the total energy dissipated by a PXCM sample can be attributed to the primary and secondary pathways. Figure S5 show respectively the *F-d* responses of the *S-* and *T- type* PXCM samples when they are

loaded at 0 degrees, for three back-to-back load-unload cycles. Irreversible (e.g. plastic) deformation across the successive cycles is negligibly small after the first cycle for all four load cases considered in this study (Table S4). This is confirmed not only by the F-d response (Fig. S5), but also by a posteriori examination of the specimens that showed no sign of permanent deformation. This is to be expected, as we designed the mechanisms such that it remained in the elastic strain regime over the complete load-unload cycle.

Estimation of the energy dissipation via the other secondary pathways listed above is not straightforward. The mechanisms are designed to have a bistable mechanical response. They exhibit snap-through behavior under force control, but not under displacement control. If we subject a single mechanism to the same range of deformation under force and displacement controlled conditions, it will dissipate energy via all available dissipation pathways in the former case. However under displacement control, it does not undergo a snap-through and, hence, it does not exhibit dissipation due to the snapping action of the mechanism. We can estimate the energy dissipated by a mechanism via all dissipation pathways except the snapping action by subtracting the energy dissipated by the mechanism under displacement control from that under force control.

Since the *S-type* PXCM loaded at {0°, 90°} showed the most irreversible deformation in successive load-unload tests, we choose that load case to estimate energy dissipation through pathways other than the snapping action of the beams. The average energy dissipated by the entire sample in cycles 2 - 3 is 6594 mJ (see Table S4). We subject a single mechanism from this sample to four successive load-unload cycles under displacement control with a cross head travel rate of 1mm /min (See Fig. S11). The energy dissipated during a complete load-unload cycle is obtained by measuring the area between the loading and unloading curve (See TableS7-S8). The average energy dissipated

by a single mechanism over cycles 2 - 4 is 17.5 mJ (see Table S8). Since, 36 such mechanisms undergo a complete load-unload cycle during a complete load-unload cycle on the entire PXCM sample (see Fig.3), we estimate the total energy dissipated by the entire sample due to pathways other than the snapping action of the beams to be 630 mJ. Thus, assuming that all 36 of these mechanisms exhibit identical energy dissipation behavior we estimate that all secondary dissipation pathways other than plastic deformation dissipate approximately 10% of the total energy dissipated by the *S-type* PXCM sample when it is loaded at 0° and 90°.

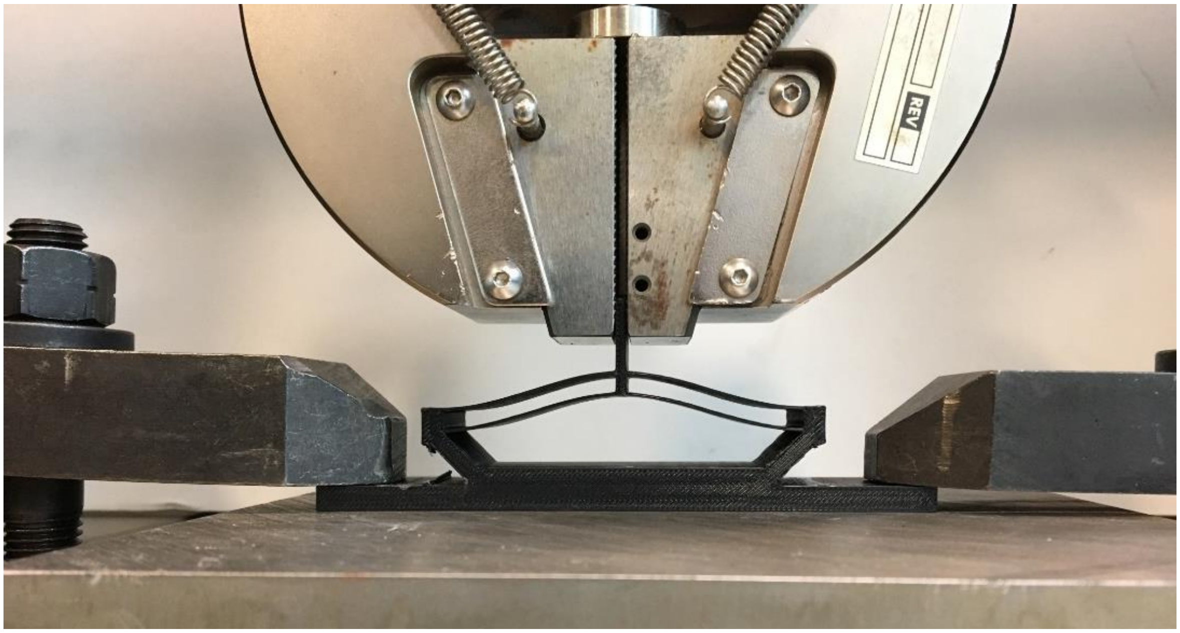

Figure S11: A single mechanism under displacement control with a crosshead travel rate of 1mm /min.

S.3.4. Auxetic behavior

Under the uniaxial loading conditions along different axes of symmetry, both *S-type* and *T-type* samples exhibit auxetic behavior from FE simulations and experiments (See Fig.3-7, and S12). These samples undergo contraction in the *X* direction when they are compressed in *Y* direction. To illustrate this effect, the Poisson's ratio ($v = -\frac{\varepsilon_Y}{\varepsilon_X}$) of *S-type* loaded at {45°,135°} and *T-type* at {30°,90°,120°} is plotted vs time in Fig. S12(b) and Fig. S12(d).

The strain in Y direction is calculated by dividing average displacement of all the nodes on top of the sample by the original sample width w: $\varepsilon_Y = \frac{\overline{u_Y}}{w}$. The strain in X direction is calculated by the subtracting the average displacement in X direction of all the nodes on left side to right side and dividing the original length of the sample L: $\varepsilon_x = \frac{\overline{u_x^r}}{L} - \frac{\overline{u_x^l}}{L}$. Based on the geometry and typology of two samples, when mechanisms fully collapsed (eq. S1), the Poisson's ratio of *S-type* at {45°, 135°} is expected to be around -1, and *T-type* at {30°,90°,120°} should be around -0.28. This analytical value and FE simulations results are plotted together at Fig. S12(b) and Fig. S12(d). It shows the good agreement between the Poisson's ratio from FE simulation and analytical results. By having these auxetic behaviors, 2D PXCMs gain more benefits such as high indentation resistance, shear modulus, fracture toughness, and synclasticity [5] . These benefits extend the application of 2D PXCMs into medical stent, adaptive clothing, and medical cast [6–8].

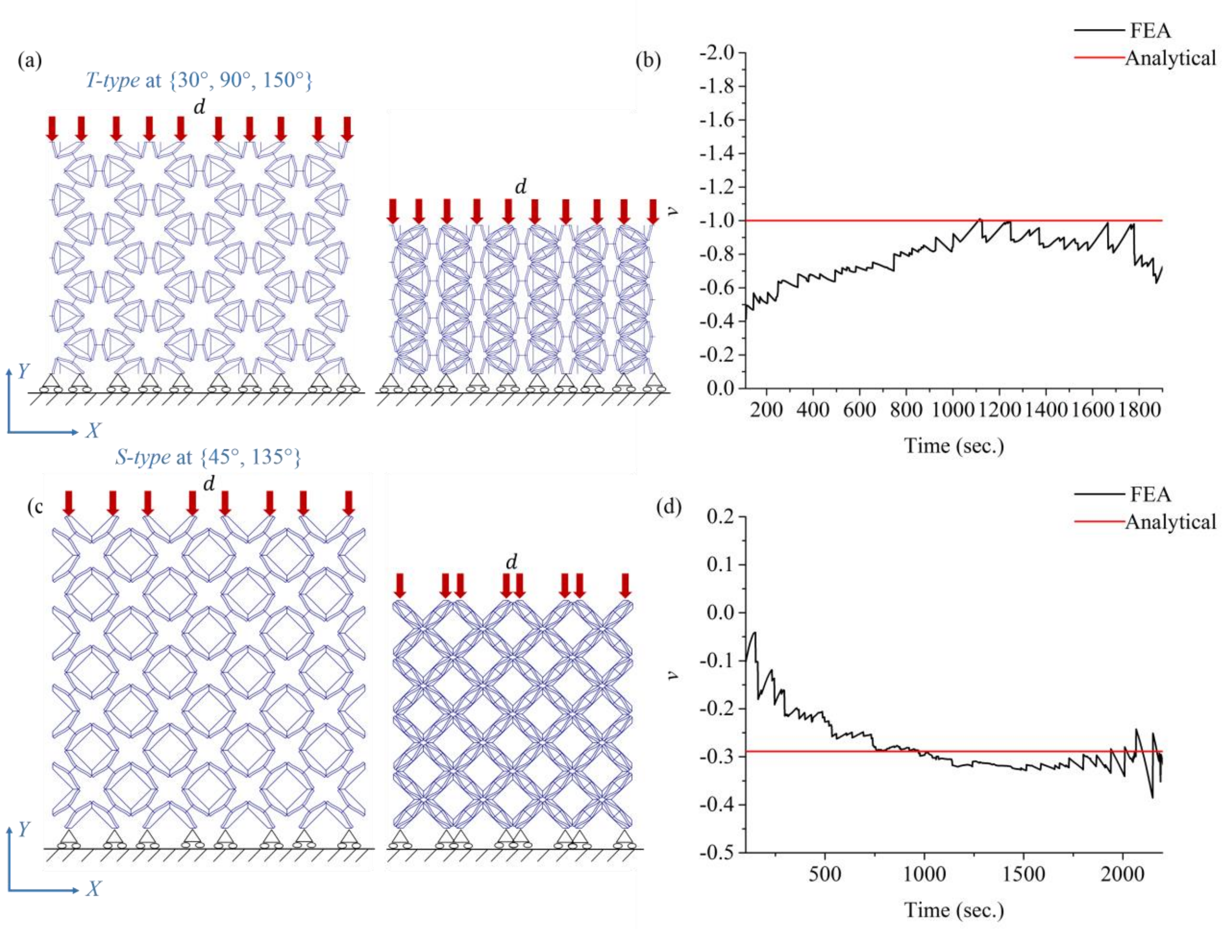

Figure S12: Poisson's ratio variation over time obtained from the numerical simulations and compared with the analytical prediction.

S4 Finite Element Simulations

Finite element models are created to quickly capture the essential mechanical response of 2D PXCMs under different loading angles. Element type, element size, contact condition, and base material properties were first studied to determine the most effective simulation set up and assess potential source of error and uncertainties.

S4.1. Element Type

To select a type of computational effective element, we create FE models of a 2D PXCM elementary mechanism (i.e. bent beam) with clamped-clamped boundary conditions and under displacement control with different elements types (Fig. S13(a)). The geometry of the bent beam is identical to the bent beams employed in the *T* and *S-type* 2D PXCMs.

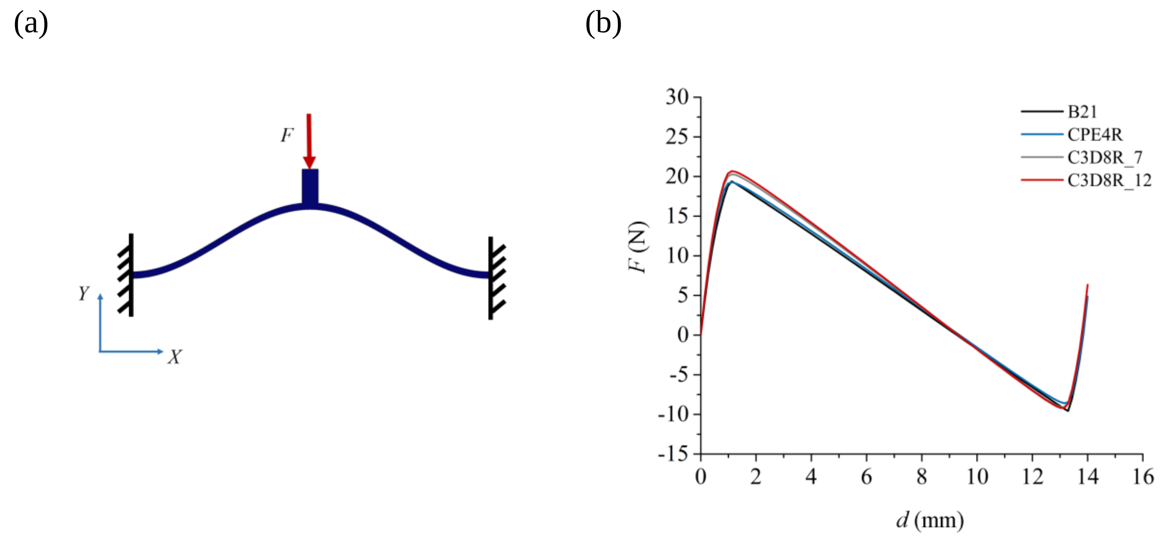

Figure S13: FE models of the elementary mechanism. (a) FE model is an elementary mechanism under the clamped-clamped boundary condition. (b) There are no significant difference can be observed by using different types of element.

The elements selected are: two-node linear beam element (B21), four-node bilinear, reduced integration with hourglass control (CPE4R) and eight-node brick element with reduced integration (C3D8R). For 2D and 3D models, 7 elements are assigned throughout the beam thickness (element size = 0.1 mm). Additionally, to check convergence for the 3D model, a model with bent beam with 12 C3D8R elements throughout the bent beam thickness is created. The *F-d* relations of four models are plotted in Fig. S13(b). As it can be observed in the figure, there is no significant difference between the models with B21, CPE4R, and C3D8R elements. We choose B21 for all

the simulations as it is the most computationally efficient and it enables us to create larger models with multiple motifs.

S4.2. Convergence study

Once the element type is chosen, a convergence study is conducted on the bent beam model with B21 elements. The element size varies from 4 mm to 2 mm. The *F-d* relation curves of five models with these various elements size are shown in Fig. S14(a). Since only minor variation can be observed from the *F-d* curves, the peak force F_p of each curve is used to quantify the difference. We plot F_p against element size as shown in Fig. S14(b). The peak load converges when the element size reduced to 2.5 mm. We chose the element size 2.17 mm which is smaller than the threshold of convergence (this is shown as red dot shown in Fig. S14(b)).

(a)

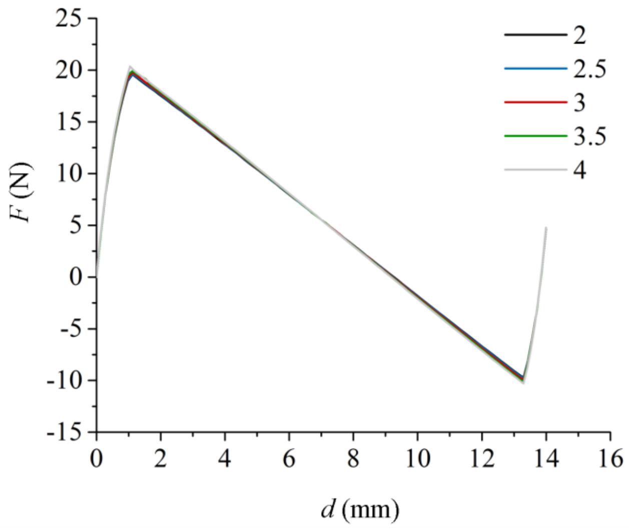

(b)

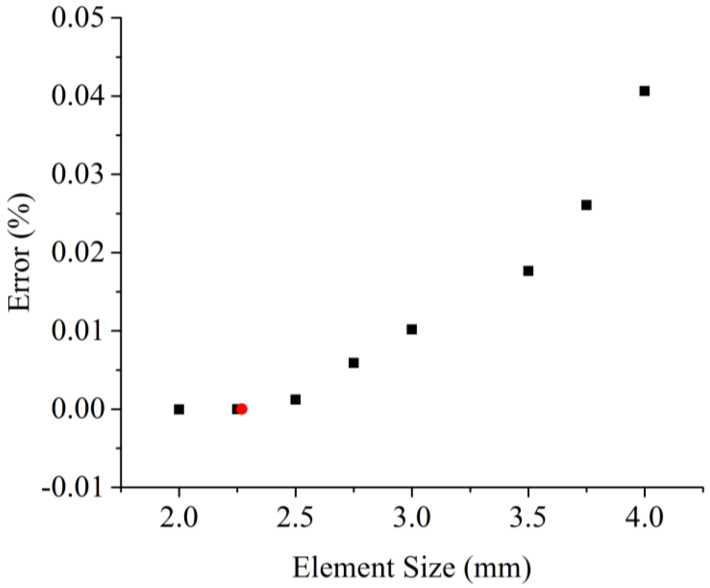

Figure S14: Convergence study of FE models. (a) *F-d* relations of FE models with element size varies from 2-4 do not show noticeable difference. (b) Peak load of bent beam starts to converge *at 2.5 mm.*

S4.3. Friction coefficient

Contact between adjacent beams was modeled using the small sliding formulation in Abaqus 6.14. Coulomb friction with a friction coefficient of $\mu = 0.1$ is assumed to be active at all contact interfaces. This value is selected based on the Typical Properties of Generic Acrylonitrile Butadiene Styrene (ABS)[9]. For generic ABS materials, the coefficient of friction varies from 0.1-0.5. To check the sensitivity of the friction coefficient in our models, we build FE models of the *S-type* PXCM loaded under {0°, 90°} (with μ varying from 0.1 to 0.5). The *F-d* relations of these models are shown in Figure S15. The model with $\mu = 0.1$ exhibits slightly lower valley force when the 11th mechanism buckles at the very end of the loading cycle. This is an indication that contact (and friction) only plays a role when most of the motifs are already transformed/collapsed. Overall, as it is evident in Fig. S15, friction seems to play a minor role in full compression and $\mu = 0.1$ is reasonable assumption to made for FE simulation.

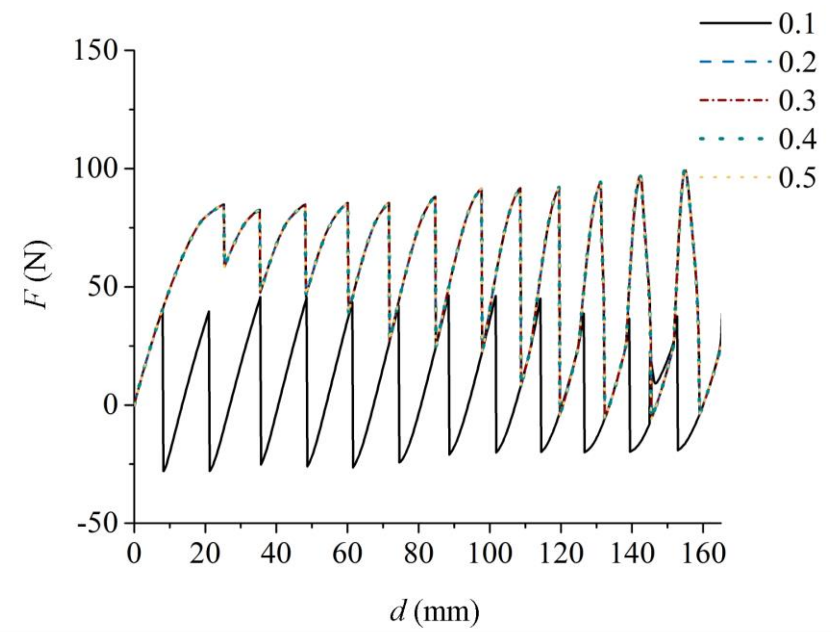

Figure S15: *F-d* relation of a *S-type* PXCM sample under the loading angle {0°, 90°} with different friction coefficient.

S 4.4. Base material properties

Three-point bending tests are conducted to determine mechanical properties of the base material. Test setup is shown in Figure S16(a). Four increasing load-unload cycles are applied on three samples as shown in Figure S16(b). Sample B1 and B2 have identical cross section areas (i.e., 0.7 mm × 25 mm) compared with the elementary mechanism of 2D PXCMs. Sample S1 also has the same thickness 0.7 mm, but half of the width (i.e. 13 mm) compared with 2D PXCMs (Table S9)

Figure S16(b) shows the *F-d* relation of sample B2 under four increasing displacement load-unload cycles.

During the first two load-unload cycles, the sample exhibits approximately linear elastic behavior. A plateau is reached when the strain increases to 0.9% and 1.2% at cycle 3 and 4 which indicates

that the base material exhibit nonlinear behavior under the large strain. However, for simplicity, we assume a linear elastic material model for the FE simulations, where the modulus is derived from the zero-strain tangent to the *F-d* curves obtained from a 3-point bending test (Table *S 10*). This simplification allows us to capture the essential mechanics of the material behavior in a computationally efficient manner, but it sacrifices the accuracy of the force prediction. This factor could cause the FE models to overestimate the mechanical response of the PXCMs compared with the experiments. To assess the uncertainties introduced by this approach we develop FE models of all four 2D PXCMs samples where we assign a minimum, average and maximum elastic modulus. Such analysis can provide lower and upper bounds on the *F-d* curves are displayed in Figure S17. The energy dissipation calculated in each sample is summarized in Table S11. Due to the uncertainty of elastic modulus of base material, assuming all the 2D PXCM samples have a constant elastic modulus can cause the energy dissipation capacity of these materials varies from -6% to 7%.

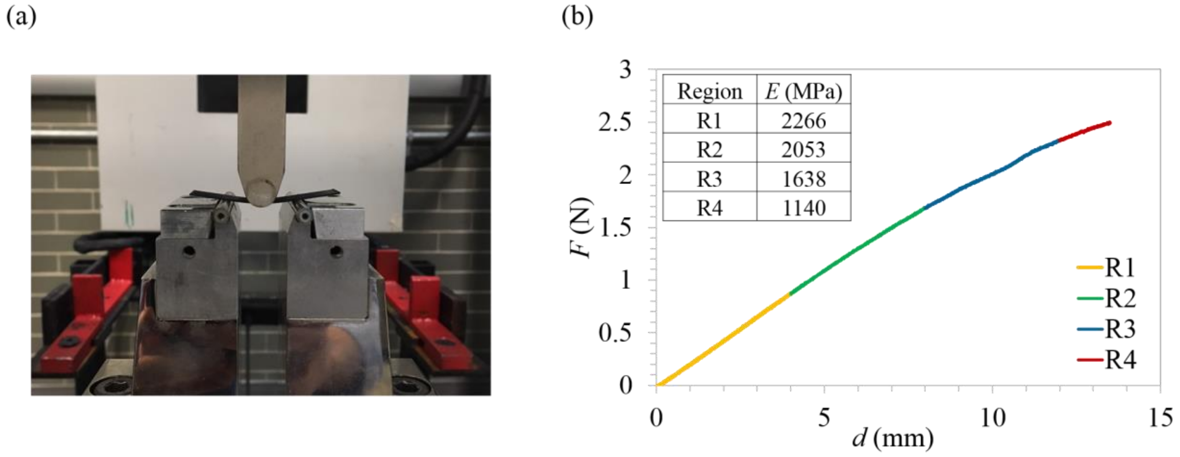

Figure S16: Three-point bending test to determine material properties of the base materials. (a) Experiment setup. (b) Applied displacement and reaction force varies with time. (c)-(f) *F-d* relation of sample under different load-unload cycles.

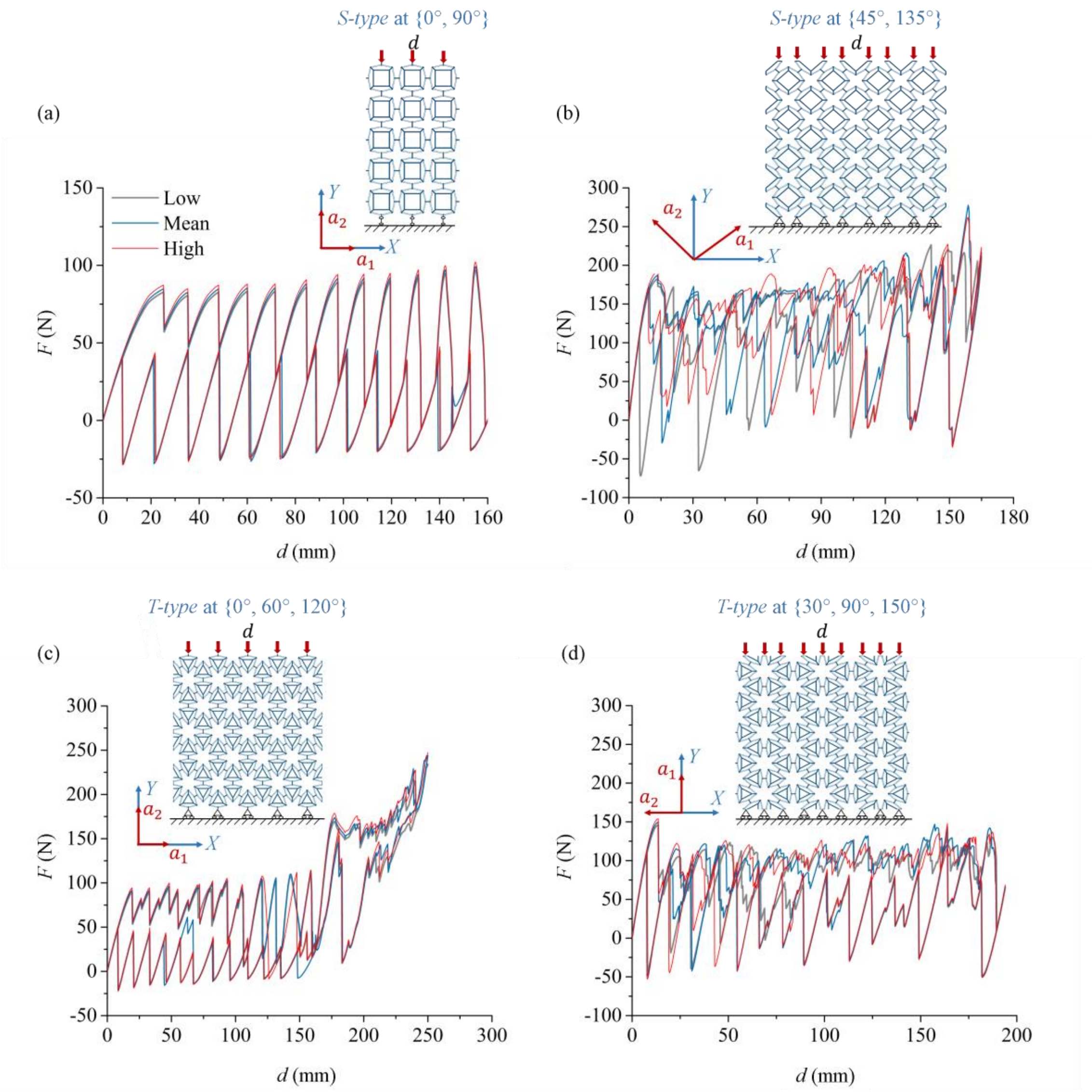

Figure S17: *F-d* relation of 2D PXCMs with minimum, average, and maximum base materials properties. (a) A *S-type* PXCM sample under three compressive load-unload cycle at {0°, 90°}. (b) A *S-type* PXCM sample under three compressive load-unload cycle at {45°, 135°}. (c) A *T-type* PXCM sample under three compressive load-unload cycle at {0°, 60°, 120°}. (d) A *T-type* PXCM sample under three compressive load-unload cycle at {30°, 90°, 150°}.

S 5. Energy dissipation rate

Energy dissipation rate is another important factor for engineering applications. To understand the how loading angle influence the energy dissipation rate in 2D PXCMs, we plot the energy dissipation for four samples as a function of the applied displacement. Energy dissipation in PXCMs occurs in a discontinuous way through discrete steps corresponding to snap through transitions in individual building blocks. However, we can define an average energy dissipation

rate in the following way: we load the material up to a snap through at a displacement and then unload it completely. As such the average energy dissipation rate, at that given displacement, can be defined as the ratio between the energy dissipated in this complete load-unload cycle and the applied displacement. This is repeated for all snap through events to get the average energy dissipation rate for different displacement values through its loading history. For this we use all the data from Figs. 3-6. These *F-d* curves can be discretized into cycles. Each cycle starts form the initial state, loads to a snap back point and then unloads back to the initial state (Figure S18(a)). The initial stiffness is used to extrapolate the unloading path after snap back happens. Figure S18(a) demonstrates the first loading and loading cycle as an example. S0 represents *S-type* PXCM under loading angle {0°, 90°}, S45 represents *S-type* PXCM under loading angle {45°, 135°}, T0 represents *T-type* PXCM under loading angle {0°, 60°, 120°}, and T90 T0 represents *T-type* PXCM under loading angle {30°, 90°, 150°}. T0 exhibits two distinguished regions therefore named by T0-1 and T0-2. Energy dissipation rate per unit volume and unit mass are used to evaluate the performance of 2D PXCMs under different loading directions (Fig. 7(c) and Figure S18(b)). Both plots show *S-type* PXCM loaded at {45°, 135°}, *T-type* PXCM loaded at {30°, 90°, 150°}, and the first region for *T-type* PXCM loaded at {0°, 60°, 120°} show the similar energy dissipation rate. *S-type* PXCM under loading angle {0°, 90°} shows higher energy dissipation rate. *T-type* PXCM is not sensitive to the loading direction compared with *S-type* PXCM. To have explicit comparison, liner interpolation is used to quantify the energy dissipation per unit value and mass varies with applied displacement. Linearized curves are shown in Figure S18 (c)-(d) and the slope of each sample is displayed in Table S12. It shows that before *T-type* PXCM loaded from {0°, 60°, 120°} enter the second region, *T-type* PXCM has identical energy dissipation rate per unit volume and mass under a load comes from any the reflectional axes of symmetry. The energy

dissipation rate for *T-type* PXCM is about two third of the one calculated for the *S-type* PXCM under at {0°, 90°}. The *S-type* at {45°, 135°}, shows 23% higher energy dissipation rate compared with the *T-type* PXCM. Once loading *T-type* PXCM along {0°, 60°, 120°} to the second region, the energy dissipation rate reduces to half. Overall, *S-type* PXCM loaded under {0°, 90°} shows highest energy dissipation rate compared to the other cases. The *S-type* at {45°, 135°} and *T-type* PXCM under all the loading angles show similar energy dissipation rate until *T-type* PXCM enters the second region under the loading direction {0°, 60°, 120°}.

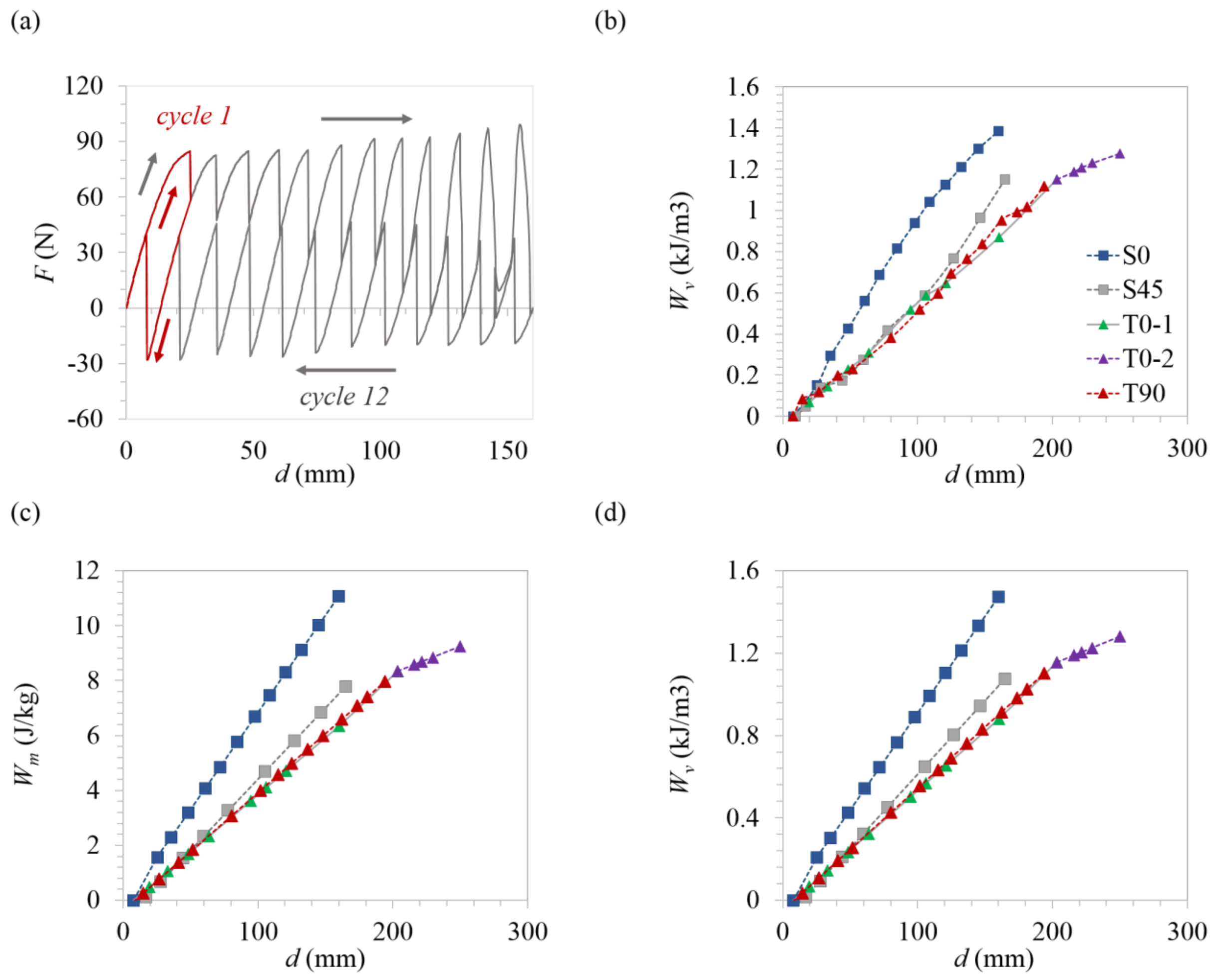

Figure S18: Energy dissipation rate of *T-type* PXCM is not sensitive to loading direction. *S-type* PXCM shows higher energy dissipation rate when load comes along the 0° compared with 45°. (a) Demonstrate energy dissipation of cycle 1 and cycle 12. (b) Energy dissipation per unit volume of four samples varies with time. (c)-(d) Energy dissipation per unit mass and per unit volume of four samples varies with time after linear curve fitting.

S 6. Biaxail loading condition

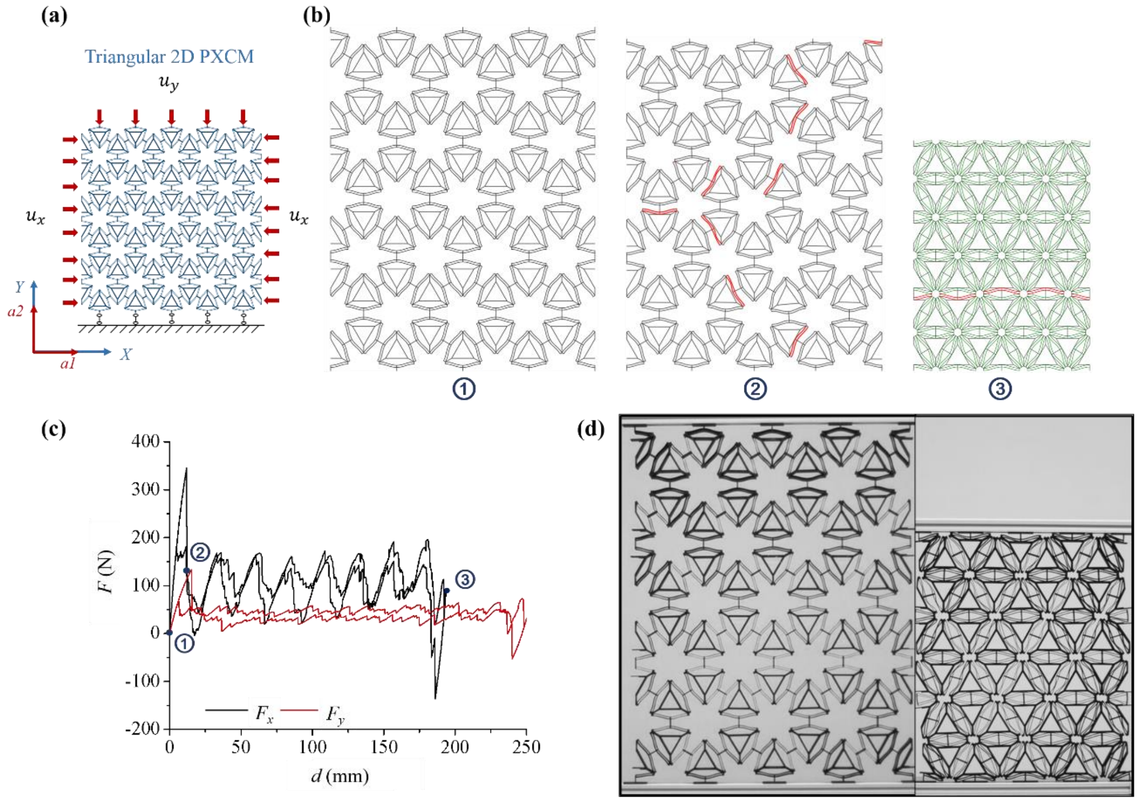

Figure S19: Performance of a *T-type* PXCM sample under one biaxial compressive load-unload cycle. (a) The sample is under uniaxial loading condition and supported by rollers at bottom. (b) Phase transformation sequence of the three characteristic states from FE simulation. (c). *F-d* relation of sample from FE simulation and Experiment. (d) Two states of the sample corresponding to initial and final deformed configurations.

We created FE simulations to investigate the response of a *T-type* PXCM under biaxial loading condition. The schematic for this load case is shown in Fig. S19(a) where the a_1 axis is aligned with the X-axis and a_2 axis is aligned with the Y-axis. To ensure all the building blocks achieve phase transformation at the same time, the loading rate in the X direction is 0.39 mm/min and in Y direction is 1 mm/min. Fig. S19(b) shows the undeformed and deformed configurations of the sample at three salient points during its compression as obtained from the finite element simulations. The corresponding points are labelled on the *F-d* response in Fig. 19(c). The bent beams are color coded according to their status at any point during the deformation process. The

beams rendered in gray are still in phase 1 (according to the definition in Design Considerations section Fig. 1 (b) , those shaded green have already transformed to phase 2, and the red ones are undergoing phase transformation.

Unlike the response of *T-type* PXCM under the uniaxial loading condition, all the building blocks undergo phase transformation under the biaxial loading condition. Expect at loading-unloading transition point, *T-type* PXCM exhibits higher serrated loading and unloading plateau force in the *X* direction than in the *Y* direction. The peak force is reduced to around 50% in both *X* and *Y* directions after the first building block transformed (See Fig. S19 (c)). The ratio of energy dissipation capacity of in *X* to *Y* direction is 1.3 (Table S13 and video 6).

Table S1. Values assigned to various design parameters in Fig. 2

Parameters	Dimension (mm)
t	0.7
t'	0.7
t_{stiff}	4.2
$t_{central}$	2.1
s	3.5
A	7
λ	71

Table S2. The volume of the undeformed the samples.

Specimen Name	Size (mm^3)
S-type 0°	6125000
S-type 45°	10400625
T-type 0°	11041875
T-type 90°	11041875

Table S3. Summary of energy dissipation of four samples under the three compressive load-unload cycles.

Specimen	Cycle	Dissipated Energy (Nmm)	Variation (%)
T-type 0°	1	15017	5%
	2	14043	-2%
	3	13894	-3%
Average		14318	
T-type 90°	1	13426	5%
	2	12689	-1%
	3	12265	-4%
Average		12793	
S-type 0°	1	7189	6%
	2	6366	-6%
	3	6823	0.5%
Average		6792	
S-type 45°	1	11016	8%
	2	10218	0.1%
	3	9402	-8%
Average		10212	

Table S4. Energy dissipation varies slightly among cycle 2 and 3.

Sample	Cycle	Dissipated Energy (Nmm)	Variation (%)
T-type 0°	2	12689	1.7%
	3	12265	-1.7%
Avg. cycle 2&3		12477	
T-type 90°	2	14043	0.5%
	3	13894	-0.5%
Avg. cycle 2&3		13969	
S-type 0°	2	6366	-3.5%
	3	6823	3.5%
Avg. cycle 2&3		6595	
S-type 45°	2	10218	4.2%
	3	9402	-4.2%
Avg. cycle 2&3		9810	

Table S5. Energy dissipation per unit volume of 2D PXCMs and 1D PXCMs along different loading angles corresponding to their axes of symmetry.

T-type PXCM				*S-type* PXCM				1D PXCM	
Experiment		FEA		*Experiment*		*FEA*		FEA	
Angle	W_v	Angle	W_v	Angle	W_v	Angle	W_v	Angle	W_v
°	kJ/m^3	°	kJ/m^3	°	kJ/m^3	°	kJ/m^3	°	kJ/m^3
0	1.27	0	1.28	0	1.08	0	1.39	0	3.32
30	1.11	30	1.12	45	0.94	45	1.15	180	3.32
60	1.27	60	1.28	90	1.08	90	1.39		
90	1.11	90	1.12	135	0.94	135	1.15		
120	1.27	120	1.28	180	1.08	180	1.39		
150	1.11	150	1.12	225	0.94	225	1.15		
180	1.27	180	1.28	270	1.08	270	1.39		
210	1.11	210	1.12	315	0.94	315	1.15		
240	1.27	240	1.28	360	1.08	360	1.39		
270	1.11	270	1.12						
300	1.27	300	1.28						
330	1.11	330	1.12						
360	1.27	360	1.28						

Table S6. Energy dissipation per unit mass of 2D PXCMs and 1D PXCMs along different loading angles corresponding to their axes of symmetry.

T-type PXCM				*S-type* PXCM				1D PXCM	
Experiment		FEA		*Experiment*		FEA		FEA	
Angle	W_m	Angle	W_m	Angle	W_m	Angle	W_m	Angle	W_m
°	J/kg	°	J/kg	°	J/kg	°	J/kg	J/kg	J/kg
0	10.49	0	9.21	0	9.49	0	10.42	0	21.60
30	9.26	30	8.07	45	7.82	45	8.34	180	21.60
60	10.49	60	9.21	90	9.49	90	10.42		
90	9.26	90	8.07	135	7.82	135	8.34		
120	10.49	120	9.21	180	9.49	180	10.42		
150	9.26	150	8.07	225	7.82	225	8.34		
180	10.49	180	9.21	270	9.49	270	10.42		
210	9.26	210	8.07	315	7.82	315	8.34		
240	10.49	240	9.21	360	9.49	360	10.42		
270	9.26	270	8.07						
300	10.49	300	9.21						

330	9.26	330	8.07						
360	10.49	360	9.21						

Table S7: The energy dissipated by the entire sample in cycles 2 – 3.

Sample 1			
Cycle	*Ed* (mJ)	*Ea* (mJ)	*Ed/Ea*
2	50.5	164.1	31%
3	21.1	130.2	16%

Table S8: The energy dissipated by the entire sample in cycles 2 – 4.

Sample 2			
Cycle	*Ed* (mJ)	*Ea* (mJ)	*Ed/Ea*
2	17.91	119.68	15%
3	17.45	117.66	15%
4	17.26	116.15	15%

Table S 9: Dimension of three-point bending test samples.

Sample	*L* (mm)	*t* (mm)	*w* (mm)
S1	24	0.7	13
B1	72	0.7	25
B2	73	0.7	25

Table S 10: Summary of three-point bending tests.

	Cycle	d_{max} (mm)	m	ε_{max}	*E_0 (MPa)*
S1	1	1.2	3.12	0.9%	2303
	2	2.4	3.16	1.8%	2333
	3	3.7	3.01	2.7%	2222
	4	4.9	3.00	3.6%	2212
B1	1	3.6	0.23	0.3%	2220
	2	7.2	0.23	0.6%	2224
	3	10.9	0.23	0.9%	2228
	4	14.5	0.23	1.2%	2228
B2	1	3.7	0.22	0.3%	2266
	2	7.3	0.22	0.6%	2316
	3	11.0	0.22	0.9%	2325
	4	14.6	0.22	1.2%	2330
Min		1.224	0.219	0.003	2212
Average		7.076	1.174	0.012	2267
Max		14.601	3.160	0.036	2333

Table S 11: Energy dissipation of four 2D PXCM samples with base material has minimum, average, and maximum elastic modulus.

Sample	Elastic Modulus	Dissipated Energy (Nmm)	Variation (%)
T-type 0°	E_{min}	13434	-1%
	E_{mean}	13729	1%
	E_{max}	13461	-1%
Average		13541	
T-type 90°	E_{min}	11067	-6%
	E_{mean}	12029	2%
	E_{max}	12379	5%
Average		11825	
S-type 0°	E_{min}	8134	-2%
	E_{mean}	8260	-1%
	E_{max}	8582	2%
Average		8325	
S-type 45°	E_{min}	13530	7%
	E_{mean}	11940	-6%
	E_{max}	12524	-1%
Average		12665	

Table S12: Energy dissipation rate *S*- and *T-type* 2D PXCMs.

Sample	Energy dissipation rate	
	per unit mass	per unit volume
S0	0.071	0.0094
S45	0.052	0.0071
T0-1	0.042	0.0058
T0-2	0.019	0.0027
T-90	0.043	0.0060

Table S13: Energy dissipation capacity of *T-type* PXCM under biaxial loading condition.

Direction	W (Nmm)	W_v (kJ/m^3)	W_m (J/kg)
X	6892.2	0.727	6.335
Y	5191.3	0.547	4.772

List of Videos

Video 1: Functionally 2D Phase Transforming Cellular Materials (PXCMs)- *S-Type* at 0 degrees

Performance of a *S-type* PXCM sample under one compressive load-unload cycle at {0°, 90°}. (a) The sample is under uniaxial loading condition and supported by rollers at bottom. (b) Phase transformation sequence of the three characteristic states from FE simulation. (c). *F-d* relation of sample from FE simulation and Experiment. (d) The states of the sample at initial and final deformed configurations.

https://youtu.be/7SSe4m_OH_w

Video 2: Functionally 2D Phase Transforming Cellular Materials (PXCMs)- *S-Type* loaded at 45 degree

Performance of a *S-type* PXCM sample under one compressive load-unload cycle at {45°, 135°}.The sample is under uniaxial loading condition and supported by rollers at bottom. Phase transformation sequence of the three characteristic states from FE simulation vs. experiments. *F-d* relation of sample from FE simulation and Experiment.

https://youtu.be/jm_lTxPArf0

Video 3: Functionally 2D Phase Transforming Cellular Materials (PXCMs)- *T-Type* loaded at 0 degree

Performance of a *T-type* PXCM sample under one compressive load-unload cycle at {0°, 60°, 120°}. The sample is under uniaxial loading condition and supported by rollers at bottom. Phase transformation sequence of the three characteristic states from FE simulation vs. Experiments. *F-d* relation of sample from FE simulation and Experiment.

https://youtu.be/LZagL31YR3g

Video 4: Functionally 2D Phase Transforming Cellular Materials (PXCMs)- *T-Type* loaded at 90 degree

Performance of a *T-type* PXCM sample under one compressive load-unload cycle at {0°, 60°, 120°}. The bent beams are color coded according to their status at that point in the deformation. The beams rendered in gray are still in phase 1, those shaded green have already transformed to phase 2, and the red ones are undergoing phase transformation.

https://youtu.be/m2gCBAmHu0Q

Video 5: Functionally 2D Phase Transforming Cellular Materials (PXCMs)- *T-Type* loaded at 90 degree

Performance of a *T-type* PXCM sample under one compressive load-unload cycle at {30°, 60°, 120°}. The sample is under uniaxial loading condition and supported by rollers at bottom. Phase transformation sequence of the three characteristic states from FE simulation vs. experiments. *F-d* relation of sample from FE simulation and Experiment.

https://youtu.be/ENMFlFrS88E

Video 6: Functionally 2D Phase Transforming Cellular Materials (PXCMs)- *T-Type* under biaxial conditions

Performance of a *T-type* PXCM sample under biaxial conditions

https://youtu.be/uZlHEX4vsOI

Video 7: Functionally 2D Phase Transforming Cellular Materials (PXCMs)- *S-Type* loaded at 0, 90, 45, and 135 degrees

Performance of a *S-type* PXCM sample under one compressive load-unload cycle at {0°, 90°} and {45°, 135°}.The sample is under uniaxial loading condition and supported by rollers at bottom. Phase transformation sequence of the three characteristic states from FE simulation vs. experiments. F-d relation of sample from FE simulation and Experiment.

https://youtu.be/TnrUo72rxkk

Video 8: Functionally 2D Phase Transforming Cellular Materials (PXCMs)- *T-Type PXCM with single bent beam as building block and spokes inside motifs loaded* at 0, 30, 60, 90, 120, and 150 degrees

Performance of a *T-type* PXCM sample under one compressive load-unload cycle at {0°, 60°, 120°} and {30°, 90°, 150°}. *T-Type PXCM with single bent beam as building block and spokes inside motifs.*

https://youtu.be/czDJqA1Pfns

References

1. Jin, Q. An Electrothermally-Actuated Bistable MEMS Relay for Power Applications. *Massachusetts Inst. Technol.* 94 (2003).

2. Qiu, J., Lang, J. H. & Slocum, A. H. a Curved Beam Bistable Mechanism Bi Stable Beam Model. **13**, 137–146 (2004).

3. Restrepo, D., Mankame, N. D. & Zavattieri, P. D. Phase transforming cellular materials. *Extrem. Mech. Lett.* **4**, 52–60 (2015).

4. Haghpanah, B., Shirazi, A., Salari-Sharif, L., Guell Izard, A. & Valdevit, L. Elastic architected materials with extreme damping capacity. *Extrem. Mech. Lett.* **17**, 56–61 (2017).

5. Saxena, K. K., Das, R. & Calius, E. P. Three Decades of Auxetics Research − Materials with Negative Poisson's Ratio: A Review. *Adv. Eng. Mater.* **18**, 1847–1870 (2016).

6. Ali, M. N., Busfield, J. J. C. & Rehman, I. U. Auxetic oesophageal stents: Structure and mechanical properties. *J. Mater. Sci. Mater. Med.* **25**, 527–553 (2014).

7. Papadopoulou, A., Laucks, J. & Tibbits, S. Auxetic materials in design and architecture. *Nat. Rev. Mater.* **2**, 17078 (2017).

8. Lakes, R. S. & Elms, K. Indentability of Conventional and Negative Poisson's Ratio Foams. *J. Compos. Mater.* **27**, 1193–1202 (1993).

9. Acrylonitrile Butadiene Styrene (ABS) Typical Properties Generic ABS | UL Prospector. Available at: https://plastics.ulprospector.com/generics/1/c/t/acrylonitrile-butadiene-styrene-abs-properties-processing.
